# The bone-biofilm interface: decoding the bidirectional crosstalk in orthopedic implant infections

**DOI:** 10.3389/fcimb.2026.1912867

**Published:** 2026-10-27

**Authors:** Hao Zhu, Zhipeng Hou, Haoxin Huang, Honghua Hu, Ligui Wan

**Affiliations:** 1 Department of Orthopedics, Yueqing Hospital of Wenzhou Medical University, Wenzhou, Zhejiang, China; 2 College of Pharmaceutical Sciences, Zhejiang University, Hangzhou, China; 3 Macquarie Medical School, Macquarie University, Sydney, NSW, Australia

**Keywords:** biofilm, bone microenvironment, host-pathogen interaction, immune evasion, orthopedic implant infection, osteolysis, persister cells, *Staphylococcus aureus*

## Abstract

Implant-associated orthopedic infections remain difficult to eradicate because biofilm persistence is coupled to progressive changes in bone and local immunity. This narrative review presents the Bone-biofilm Interface (BBI) as an integrative framework linking microbial biofilms, implant properties, bone cells, immune cells, and the mineralized matrix. Its contribution is primarily organizational: it consolidates evidence on intracellular bacterial reservoirs, inflammatory osteolysis, immune dysfunction, and biofilm adaptation within a single spatial and functional unit. The framework also advances testable, but not yet clinically established, hypotheses concerning ion-mediated matrix remodeling, the dual role of osteoclasts as resorptive cells and potential bacterial reservoirs, and niche-dependent transitions between dormancy and resuscitation. We further consider how polymicrobial communities, temporal evolution, and implant material properties may modify these interactions. The available evidence is weighted according to source, with human observations distinguished from animal and *in vitro* studies. Established clinical management, including surgical source control and guideline-based antimicrobial therapy, remains central; proposed approaches involving microenvironment modulation, metabolic interference, immune reprogramming, anti-resorptive agents, and responsive biomaterials should be viewed as investigational adjuncts. By clarifying evidence boundaries and translational readiness, the BBI framework provides a structured research agenda for testing how disruption of host-microbe crosstalk might complement current treatment of chronic implant-associated infection.

## 1 Introduction

Orthopedic implants have transformed the management of trauma, degenerative joint disease, and spinal disorders, but they also create a surface and tissue environment in which pathogens can persist (Greco et al., 2025; Johnson et al., 2025). Implant-associated infection is a serious complication that prolongs hospitalization, increases costs and morbidity, and may require repeated revision surgery or amputation (Javed et al., 2023). Many periprosthetic joint infections (PJI) and fracture-related infections (FRI) can be successfully managed when they are recognized promptly and treated according to established guidelines, including appropriate debridement, implant retention or exchange when indicated, and targeted antimicrobial therapy (Tschudin-Sutter et al., 2016). Nevertheless, late presentation, compromised host factors, mature biofilm, and persistent bacterial phenotypes continue to make a clinically important subset difficult to eradicate. These infections may recur after apparently successful treatment and can remain locally destructive even when systemic inflammatory markers have normalized. With increasing arthroplasty volumes and an aging population, their absolute burden is expected to rise (Jiang et al., 2020).

A biofilm is an organized, surface-associated microbial community whose cells are embedded in a self-produced extracellular polymeric substance (EPS) matrix. The matrix contains polysaccharides, proteins, lipids, and extracellular DNA (eDNA), which is DNA released through secretion or cell lysis that contributes to adhesion, structural cohesion, nutrient retention, and horizontal gene transfer. Biofilm development is operationally described as reversible attachment, irreversible adhesion, microcolony formation, maturation with chemical and metabolic gradients, and dispersal. Quorum sensing (QS) is density-dependent chemical communication through secreted autoinducers that coordinates group behaviors, including matrix production, virulence, and dispersal, although its contribution varies among species and environmental conditions (Rutherford and Bassler, 2012; Rather et al., 2021; Dong et al., 2022; Funari and Shen, 2022; Su et al., 2022; Winans et al., 2022; Zhao et al., 2023; Markowska et al., 2024).

Functionally, the biofilm state is defined not only by the presence of matrix but also by spatially heterogeneous growth, altered gene expression, collective stress responses, and reduced susceptibility to antimicrobials and phagocytic clearance. EPS and eDNA can impede immune recognition and engulfment, whereas oxygen and nutrient gradients generate slow-growing and persister subpopulations. This tolerance should not be attributed indiscriminately to poor antibiotic penetration. Direct measurements show that vancomycin and several other agents can penetrate *Staphylococcus epidermidis* (*S. epidermidis*) biofilms on infected prostheses, while treatment failure reflects, to a large extent, the altered physiology and reduced susceptibility of embedded bacteria (Darouiche et al., 1994). The surface-biofilm model therefore explains only part of the disease. Infection extends into surrounding tissue and bone, bacteria can persist within bone cells, and periprosthetic osteolysis reflects sustained interactions among microbial products, host inflammation, and bone remodeling (Yao et al., 2025).

We define the Bone-biofilm Interface (BBI) as the spatially and functionally integrated zone in which the implant surface, microbial biofilm, bone cells, immune cells, and mineralized matrix exchange signals and metabolites (Mirzaei et al., 2024). The BBI is dynamic rather than anatomical: its composition and therapeutic accessibility change over time and are influenced by implant material properties. Within this zone, infection and inflammation generate hypoxia, acidosis, altered ion concentrations, tissue damage, and immune dysfunction. Bone resorption is executed by host effectors, including phagocyte-derived proteases and RANKL-driven osteoclasts, while persistent microbial stimulation sustains these processes. Matrix degradation may then release substrates and signals that further support bacterial persistence. The model is based mainly on monomicrobial *Staphylococcus aureus* (*S. aureus*) systems, but clinically important polymicrobial communities may alter the strength and direction of each interaction.

Osteoclasts are specialized multinucleated bone-resorbing cells derived from monocyte-macrophage precursors. Under physiological conditions, osteoclast resorption is coupled to osteoblast-mediated bone formation and is essential for skeletal remodeling, repair, and mineral homeostasis. In infection, the relevant abnormality is not the existence of osteoclast activity itself but its excessive or prolonged activation and uncoupling from bone formation through RANKL and inflammatory signaling (Siddiqui and Partridge, 2016; Tsukasaki and Takayanagi, 2019).

The BBI framework makes three distinct contributions. First, it is synthetic: it organizes established observations, including intracellular bacterial persistence, inflammatory osteolysis, immune dysregulation, and biofilm adaptation, within one spatially defined interface (**Table 1**). Second, it identifies mechanistic propositions that require direct testing. These include ion-mediated remodeling or biomineralization of the biofilm matrix, the possibility that RANKL-differentiated osteoclasts couple resorption to intracellular bacterial persistence, and a niche-dependent continuum between bacterial dormancy and resuscitation. Third, it generates falsifiable questions. For example, experiments can test whether modulation of local Ca^2+^ and inorganic phosphate (P_i_) changes matrix mechanics or susceptibility to selected antimicrobials, whether inhibition of excessive osteoclast activity reduces both bone loss and bacterial reservoirs, and whether correction of acidosis or hypoxia alters persister resuscitation or myeloid-cell function. These are research hypotheses rather than established therapeutic claims.

**Table 1:** Bidirectional signaling mechanisms at the BBI.

Direction	Key mediators	Effects on target	Pathological outcomes	References
Biofilm to Bone	Bacterial toxins; metabolic acids; PAMPs such as peptidoglycan and lipopolysaccharide; biofilm matrix components	Osteoclast activation is associated with FcγR-TLR2 crosstalk, RANKL/OPG dysregulation, and NF-κB/NFATc1 signaling; osteoblast viability and differentiation are impaired; osteocytes may be invaded and can upregulate Sost and RANKL.	Periprosthetic osteolysis, impaired bone formation, implant loosening, establishment of intracellular bacterial reservoirs.	(Widaa et al., 2012; Josse et al., 2015; Negishi-Koga et al., 2015; Mendoza Bertelli et al., 2016; Ren et al., 2017; Wang et al., 2018; Krauss et al., 2019; Tsukasaki and Takayanagi, 2019; Mouton et al., 2021; Hu et al., 2022; Yoshimoto et al., 2022; Jiao et al., 2023; Campbell et al., 2024; Qin et al., 2024; Yao et al., 2024; Rong et al., 2025; Cai et al., 2026; Crepin et al., 2026)
Bone to Biofilm	Ca^2+^ and Pi ions from hydroxyapatite dissolution; hypoxic (<1% O_2_) and acidic (pH 5.0-6.5) conditions; collagen peptides and amino acids; M2-polarized macrophages; MDSCs	Bacteria adopt persister phenotypes; SCVs emerge under chronic antibiotic exposure and are stabilized in biofilms; metabolic shift to anaerobic pathways; QS reprogramming; EPS production increases with Ca^2+^-mediated cross-linking and mineralization; alternative nutrient sources are utilized.	Antimicrobial tolerance in experimental models; immune evasion; biofilm persistence; recurrence risk.	(Guan and Liu, 2020; Rahbari et al., 2021; Soe et al., 2021; Peyrusson et al., 2022; Sathe et al., 2023; Simanek et al., 2023; Gunn et al., 2024; Luo et al., 2024; Mitroulis et al., 2024; Bhagwat et al., 2025; He et al., 2026)

PAMPs, pathogen-associated molecular patterns; FcγR, Fc gamma receptor; TLR2, Toll-like receptor 2; Sost, sclerostin; RANKL, receptor activator of nuclear factor-κB ligand; OPG, osteoprotegerin; NF-κB, nuclear factor-κB; NFATc1, nuclear factor of activated T cells cytoplasmic 1; TNFR1, tumor necrosis factor receptor 1; Ca^2+^, calcium ion; Pi, inorganic phosphate; MDSCs, myeloid-derived suppressor cells; SCV, small-colony variant; QS, quorum sensing; EPS, extracellular polymeric substances.

This narrative review synthesizes evidence from microbiology, bone biology, immunology, and biomaterials research. It does not constitute a formal systematic review. We selected mechanistic consensus, pivotal primary studies, and emerging findings to examine pathogen profiles, biofilm formation and antimicrobial tolerance, infection-driven remodeling of bone, bacterial adaptation, immune dysregulation, and the clinical implications of their interaction. The final sections assess diagnostic and therapeutic concepts while retaining established surgery and antimicrobial therapy as the clinical foundation.

Evidence is interpreted according to its source and translational relevance. Human clinical observations are distinguished from animal models and *in vitro* studies, and mechanisms supported only by preclinical work are identified as such. Neutrophil granulocytes are the principal effector cells responsible for direct bacterial killing at the implant interface, with macrophages providing additional clearance; T cells mainly regulate and sustain these innate responses rather than directly eliminating bacteria. **Figure 1** and the corresponding text have been aligned with this effector hierarchy.

**Figure 1:**
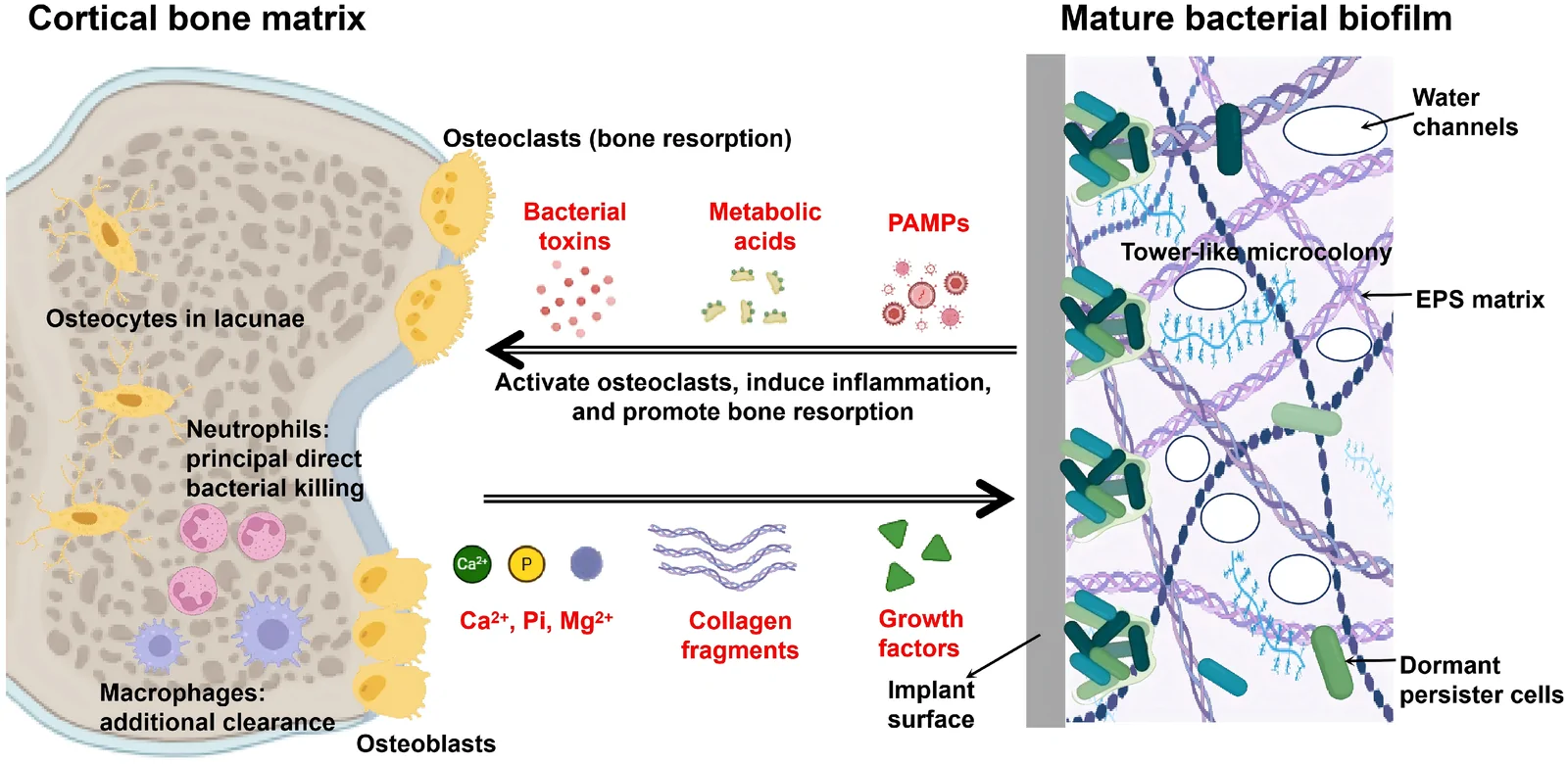
Bidirectional crosstalk at the bone-biofilm interface. A mature bacterial biofilm attached to the implant surface releases pathogen-associated molecular patterns, toxins and metabolic acids that promote local inflammation, osteoclast activation and bone resorption. Neutrophils serve as the principal direct bacterial-killing effectors, whereas macrophages provide additional clearance. Bone resorption and tissue injury alter oxygen tension and pH and release Ca^2+^, inorganic phosphate, Mg^2+^, collagen fragments and matrix-associated growth factors. These products may, in turn, influence biofilm architecture, metabolism and persistence. The effects of bone-derived ions and matrix products on biofilm fortification are context dependent and supported predominantly by preclinical evidence. EPS, extracellular polymeric substance; PAMPs, pathogen-associated molecular patterns; P_i_, inorganic phosphate.

## 2 Epidemiology and pathogen profile of postoperative orthopedic infections

### 2.1 Current status of orthopedic infection

Orthopedic surgery is inherently traumatic. Procedures typically require incising or dissecting damaged tissues, then implanting fixation devices at the injury site (Liang et al., 2024). Minimally invasive techniques and internal fixation materials have achieved remarkable breakthroughs (Li et al., 2023). Surgical outcomes have improved substantially. Yet trauma remains intrinsic to these operations. Pathogenic organisms abound in clinical environments. Consequently, postoperative infection continues as a frequent complication despite technological advances.

Reported infection rates vary substantially across procedures, case definitions, and clinical settings. Implant-associated infection occurs in approximately 1-3% of many orthopedic procedures but is considerably more frequent after severe open fractures (Pigeolet et al., 2024). In a 2019 multicenter study from southern China, extremity post-traumatic osteomyelitis was associated with a median hospitalization cost of $10,504, nearly five times that of uninfected controls (Jiang et al., 2020). External fixation accounted for most material expenditure, and open-FRI increased costs relative to closed fractures. The burden also includes prolonged hospitalization, extended follow-up, loss of work capacity, and associations with systemic and psychological comorbidity (Panteli and Giannoudis, 2016; Alalman et al., 2024; Yang et al., 2025). These data support rigorous perioperative prevention and timely, guideline-based management.

### 2.2 Distribution characteristics of pathogenic microflora in orthopedic infections

Postoperative incision infection, driven by factors such as soft tissue trauma, internal fixation devices, and prolonged surgery, imposes substantial economic and clinical burdens on orthopedic patients, occasionally necessitating amputation or threatening life (Ogihara et al., 2021; Liu et al., 2025). Conventionally, perioperative prophylactic antimicrobial agents are administered prior to incision to prevent surgical site infection (SSI). Inadequate agent selection, incorrect timing, or unnecessary prolongation of prophylaxis into the postoperative period risks fostering drug-resistant bacterial strains and may trigger shifts in pathogenic microbial profiles, thereby complicating clinical management. A further complicating factor is that orthopedic implant-associated infection encompasses heterogeneous clinical entities, including PJI, FRI, internal fixation-associated infection, and deep SSI. These categories differ in pathogen distribution, temporal patterns, and clinical outcomes, and data from one type should not be uncritically applied to another. Consequently, analyzing the distribution characteristics of pathogenic microorganisms within specific infection types becomes essential for rational antimicrobial use in postoperative infection control.

Traditional perspectives identify Gram-positive cocci as the predominant pathogens in orthopedic infections, with *S. aureus* and coagulase-negative staphylococci (CNS) standing out prominently. Yet the relative contribution of these organisms varies markedly by infection type. These organisms constitute a major etiological force, accounting for 41-58% of orthopedic infections across various studies, led specifically by *S. aureus* and *S. epidermidis* (Tschudin-Sutter et al., 2016). Most orthopedic procedures involve implantation of foreign materials ranging from prosthetic joints to titanium mesh bone grafts, and are typically performed under general anesthesia requiring endotracheal intubation and urinary catheterization. Such invasive medical devices expose the body to heightened infection risks (Pietrocola et al., 2022). In one large-scale investigation covering 359,268 orthopedic surgical patients, *S. aureus* infection occurred at a rate of 1.13%, emerging as the most frequently isolated microorganism from postoperative incision infections (Dreyfus et al., 2022). Kuehl et al. (2019) prospectively followed 229 patients with internal fixation-associated infections over a decade and similarly identified *S. aureus* as the most prevalent pathogen at 41.9%, validating its dominant role across diverse orthopedic settings.

In FRI, whether microbial distribution varies with time since injury has been examined directly, and the evidence does not support a systematic temporal shift in infecting agents. In a large multicenter cohort, Corrigan et al. found that the causative pathogens did not differ significantly between early, delayed, and late FRI (Corrigan et al., 2022). Prospective data from Kuehl et al. similarly identified *S. aureus* as the most frequent pathogen across all three time periods, at 41.9% overall, with CNS the only organisms significantly more frequent in delayed and late infections than in early ones (Kuehl et al., 2019). Time since injury nonetheless remains clinically decisive: implant retention succeeds in 85.7% of early cases but falls to 9.8% in late infections, underscoring the urgency of early intervention (Kuehl et al., 2019).

Microbiological distributions differ among PJI, FRI, and deep SSI. PJI is commonly monomicrobial and dominated by *S. aureus* and CNS, whereas polymicrobial infection is more frequent after open fractures (Tschudin-Sutter et al., 2016; Corrigan et al., 2022). Regional SSI cohorts may show a larger proportion of Gram-negative organisms. For example, Das et al. reported Gram-negative bacteria in 74.2% of postoperative orthopedic SSI, whereas Kim et al. found that deep SSI was predominantly caused by Gram-positive cocci, with *S. aureus* accounting for 45.7% (Kim et al., 2024; Das et al., 2025). *Pseudomonas aeruginosa* (*P. aeruginosa*) is therefore relevant in selected settings but should not be presented as a common FRI pathogen; a large referral-center series attributed only 5.5% of implant-associated bone and joint infections to this organism (Sathe et al., 2023). These estimates are context dependent and may also underestimate diversity because culture-negative infection remains common.

## 3 Bacterial biofilm formation and antimicrobial resistance mechanisms

Bacterial biofilms are structured microbial communities encased in EPS or exopolysaccharides, exhibiting unique three-dimensional architectures. These polymicrobial communities comprise not only bacteria but also extracellular DNA (eDNA), proteins, viruses, fungi, and other biological factors (Funari and Shen, 2022). Bacterial biofilms attached to surfaces provide protective effects, enabling bacteria to withstand harsh environmental conditions, resist antimicrobial agents, enhance survival against phagocytic cells, and evade host immune responses, thereby increasing bacterial viability (Lv et al., 2023). While the term “infections associated with bacterial biofilms” is often used broadly, it is important to note that biofilm involvement varies by infection type; for instance, PJI are most commonly monomicrobial with biofilm formation on the implant surface, whereas open FRIs more frequently present as polymicrobial communities (Tschudin-Sutter et al., 2016; Corrigan et al., 2022).

### 3.1 Molecular mechanisms underlying bacterial biofilm formation

Biofilm formation is a dynamic, multistep process initiated when bacteria reversibly attach to abiotic or biotic surfaces through non-specific physicochemical interactions, including van der Waals forces, electrostatic interactions, and hydrophobic effects (Rather et al., 2021). This initial reversible attachment subsequently transitions to irreversible adherence mediated by specific bacterial surface adhesins and the secretion of EPS and exopolysaccharides. The secretion of EPS, proteins, and eDNA is characteristic of the accumulation and maturation phases, rather than the initial attachment phase. Following stable attachment, proliferating bacteria aggregate into microcolonies that undergo further growth and differentiation, ultimately developing into mature biofilm communities characterized by towering cell clusters and intricate water channel architectures. These specialized structures facilitate nutrient delivery to deeper biofilm layers while enabling efficient removal of metabolic waste. Upon maturation, biofilm communities enter a dispersal phase where bacteria undergo passive or active detachment to colonize new surfaces (Su et al., 2022). The extracellular polymeric matrix serves as the structural foundation of biofilm communities and performs multiple critical functions, most notably mediating robust surface attachment and forming a protective barrier that shields resident bacteria from antibiotic killing and host immune clearance (Zhao et al., 2023). Mature biofilms typically display distinctive architectural features including water channels and mushroom-shaped macrocolonies that maintain internal homeostasis through selective transport processes (Winans et al., 2022). The developmental and physiological coordination of biofilm communities depends on quorum sensing (QS)-mediated intercellular communication, which drives both biofilm maturation and virulence factor expression (Markowska et al., 2024). As a key regulator of bacterial phenotypic switching, QS triggers extensive genetic reprogramming in planktonic bacteria, inducing novel protein expression patterns that promote the transition to sessile biofilm lifestyles (Rutherford and Bassler, 2012). Spatial heterogeneity within biofilm architecture generates distinct microenvironments where oxygen availability and metabolic status vary considerably with bacterial positioning, creating localized zones of genetic and physiological diversification. These gradients of hypoxia and differential metabolic activity within biofilm interiors constitute fundamental determinants of the heightened antimicrobial resistance characteristic of biofilm-associated infections (Jin and Riedel-Kruse, 2024).

In polymicrobial infection, colonization can be sequential rather than simultaneous. Early colonizers first adhere to the implant or host-protein conditioning film and modify the surface through matrix deposition and oxygen consumption. Secondary organisms can then co-aggregate with the primary colonizers or bind to shared EPS, while metabolic cross-feeding, altered redox gradients, and interspecies signaling stabilize the consortium. These interactions may also include competition, so the dominant species can change during treatment or dispersal. The resulting community is therefore shaped by arrival order, material surface, local nutrients, antimicrobial exposure, and host immunity rather than by species identity alone (Gabrilska and Rumbaugh, 2015; Dhamgaye et al., 2016; Abedon, 2019).

### 3.2 Mechanisms of antimicrobial resistance in bacterial biofilms

Compared with planktonic bacteria, biofilm-embedded bacteria can show markedly increased tolerance in experimental assays. For example, *S. aureus* biofilms were 50-fold more resistant to sodium hypochlorite and 600-fold more resistant to benzalkonium chloride in an *in vitro* disinfectant study (Luppens et al., 2002). In a separate rodent femoral implant-infection study, Okae et al. measured minimum biofilm eradication concentrations directly from explanted, *in vivo*-grown biofilms and found that conventional antibiotics generally required concentrations within one to two orders of magnitude above planktonic values (Okae et al., 2022). These model-specific results should not be extrapolated directly to clinical efficacy. Likewise, planktonic *P. aeruginosa* failed to survive 50 μg/mL tobramycin in an experimental system, whereas biofilm-resident bacteria tolerated concentrations up to 1 mg/mL (Okae et al., 2022). Collectively, these findings illustrate biofilm-associated tolerance while emphasizing that its magnitude depends on the organism, agent, model, and endpoint.

Biofilm-associated antimicrobial tolerance reflects several interacting mechanisms. Early models emphasized restricted diffusion through the EPS matrix, but penetration is agent and matrix dependent rather than uniformly impaired. In infected prostheses, vancomycin concentrations within *S. epidermidis* biofilm exceeded the MIC and MBC, yet bacteria persisted because their physiological state reduced susceptibility (Darouiche et al., 1994). Charged matrix components can still sequester selected agents, while nutrient and oxygen gradients promote slow growth, stress responses, and persister formation (Shrestha et al., 2022). Genetic resistance can additionally spread through horizontal gene transfer facilitated by close cellular proximity and eDNA (Shrestha et al., 2022).

EPS should not be viewed as material that phagocytes simply ingest and remove as an intact structure. Matrix polysaccharides, eDNA, and associated proteins can mask opsonic targets, increase the effective size of bacterial aggregates, and restrict neutrophil or macrophage contact with embedded cells. Direct experiments with *S. epidermidis* show that eDNA and polysaccharide intercellular adhesin protect mature biofilms from phagocytosis by polymorphonuclear neutrophils (Evans et al., 2025). Phagocytes can ingest detached bacteria and small matrix-associated fragments, but engulfment of an intact mature biofilm is inefficient and may lead to persistent, tissue-damaging inflammation (Zimmerli et al., 1984; Evans et al., 2025).

The antimicrobial resistance mechanisms of bacterial biofilms operate through multiple interconnected pathways. One major contributor involves the upregulation of bacterial efflux pump systems that actively extrude antibiotics from bacterial cells. Research has demonstrated that the classic efflux pump genes PA1874–1877 in *P. aeruginosa* are significantly upregulated within biofilms, thereby increasing resistance to ciprofloxacin, gentamicin, and tobramycin (Zhang and Mah, 2008). Concurrently, the biofilm matrix itself can impair the penetration of specific antibiotics through physicochemical sequestration mechanisms. eDNA and charged polysaccharides can sequester certain antibiotics and impede their diffusion through the matrix, though this inhibitory effect exhibits selectivity; for example, Klebsiella pneumoniae biofilms demonstrate weak inhibition of ciprofloxacin diffusion but markedly stronger restriction of ampicillin penetration (Anderl et al., 2000).

The extracellular matrix additionally serves as a hotspot for horizontal gene transfer, a critical driver of genetic antimicrobial resistance dissemination within biofilm communities. Bacteriophages, viruses, and plasmids function as vectors for resistance gene transmission, although some eDNA can also be transferred to recipient cells independent of carrier mechanisms (Shrestha et al., 2022). Research has reported that *S. aureus* biofilms facilitate horizontal transfer of plasmid-borne antibiotic resistance genes through conjugation and transformation processes (Savage et al., 2013). These genetic mechanisms yield heritable and transferable resistance traits that persist irrespective of biofilm dispersal, in contrast to the reversible phenotypic protection afforded by matrix sequestration alone.

## 4 Remodeling of the host terrain: infection-driven alterations of the bone microenvironment

### 4.1 Hypoxia and acidosis: shared byproducts of inflammation and bacterial metabolism

The bone microenvironment at an infected implant site undergoes a profound metabolic shift, characterized by the development of severe hypoxia and acidosis. This altered landscape is a synergistic creation. The host’s inflammatory response features massive infiltration of neutrophils and macrophages. These cells consume oxygen at a high rate through the respiratory burst. Meanwhile, the dense biofilm matrix and necrotic tissue physically impede oxygen diffusion (He et al., 2026). Concurrently, bacterial metabolism, particularly the shift to fermentative pathways like pyruvate and arginine fermentation under oxygen limitation, generates substantial amounts of short-chain fatty acids and carbon dioxide (CO_2_), directly acidifying the local environment (Bhagwat et al., 2025). This co-created state of hypoxia and acidosis is not merely a bystander effect but a powerful driver of infection chronicity, acting as a master regulator of both bacterial physiology and host response mechanisms.

#### 4.1.1 Driving bacterial phenotypic switching and QS reprogramming

The hypoxic and acidic conditions within the BBI serve as potent environmental cues that trigger fundamental reprogramming of bacterial physiology, enhancing fitness and treatment resistance. *P. aeruginosa*, although uncommon in fracture related infection and accounting for only 5.5% of implant-associated bone and joint infections in a large reference center series (Winans et al., 2022), exemplifies this adaptive prowess when it is present; we therefore use it here as a mechanistic exemplar of hypoxia driven reprogramming rather than as a representative FRI pathogen. Under oxygen limitation, it undergoes a metabolic shift from aerobic respiration to anaerobic pathways. These include denitrification and fermentation. These processes are governed by the master transcriptional regulators: anaerobic nitrate respiration regulator (ANR) and dissimilative nitrate respiration regulator (DNR) (Sathe et al., 2023). This metabolic reprogramming is intrinsically linked to increased antibiotic tolerance. Furthermore, hypoxia directly activates key virulence determinants, notably the type III secretion system (T3SS), through regulators like ANR and the glyoxylate shunt enzyme isocitrate lyase (ICL) (Cerioli et al., 2020). The acidic microenvironment synergistically reinforces this pathogenic adaptation.

Critically, hypoxia and acidosis remodel bacterial cell-to-cell communication (Hong et al., 2019). QS systems, which coordinate population-level behaviors like biofilm formation and virulence factor production, are significantly influenced by these conditions (Liu et al., 2025). For instance, the expression of lasI and rhlI autoinducer synthases in *P. aeruginosa* is upregulated under low oxygen, potentially hyper-activating QS circuits (Simanek et al., 2023). Simultaneously, the accumulation of fermentation byproducts and the altered redox state can influence the stability, diffusion, or perception of QS signal molecules. This hypoxia/acidosis-driven reprogramming of QS and metabolism converges on the secondary messenger cyclic di-GMP (c-di-GMP), a central switch for the biofilm lifestyle. The resulting sustained high intracellular c-di-GMP levels reinforce biofilm stability, repress motility, and enhance matrix production, cementing the bacteria in a dormant, treatment-resistant state (Simanek et al., 2023). In essence, the hostile host terrain paradoxically selects for and sustains the most resilient and dangerous bacterial phenotypes.

#### 4.1.2 Impairing antibiotic activity and immune cell function

The same hypoxic and acidic microenvironment that stabilizes the biofilm creates a dual barrier that severely compromises both antimicrobial therapy and host immunity (Li et al., 2024). From a pharmacological perspective, the efficacy of many first-line antibiotics is critically dependent on bacterial metabolic activity and growth (Alaoui Mdarhri et al., 2022). Hypoxia-induced metabolic dormancy and the slow-growing or persister cell states within the biofilm core render these cells inherently tolerant to antibiotics that target active cellular processes (Wainwright et al., 2021). Additionally, acidosis can directly alter the chemical state or binding affinity of certain antibiotic classes, reducing their antimicrobial potential (Guan and Liu, 2020). The acidic pH may also impair the activity of aminoglycosides by reducing their uptake into bacterial cells.

Perhaps more concerning is the compromising effect of hypoxia and acidosis on the effector cells of the innate immune system. Neutrophils and macrophages, crucial for phagocytosing and killing pathogens, are functionally impaired in this milieu. Hypoxia disrupts the oxidative burst, the primary microbicidal weapon of phagocytes, by limiting the substrate required for reactive oxygen species (ROS) generation (Guan and Liu, 2020). Furthermore, the low pH and lactate accumulation can inhibit phagocytic chemotaxis, engulfment, and intracellular killing mechanisms. Macrophages may be polarized towards a pro-inflammatory, tissue-damaging M1 phenotype initially, but chronic hypoxia often leads to dysfunctional or immunosuppressive states, impairing clearance (Luo et al., 2024). This creates a notable paradox. The host’s inflammatory response contributes to creating conditions that disable its own frontline defenders. This allows the biofilm to endure unchallenged while collateral damage to bone tissue continues.

### 4.2 Disruption of ionic homeostasis: the “mineral storm” unleashed by bone resorption

Bone resorption alters the ionic environment of the infected interface. Dissolution of hydroxyapatite by osteoclast activity and local acidity releases Ca^2+^, P_i_, and Mg^2+^ (Rozis et al., 2021; Terkawi et al., 2022). These ions may influence biofilm architecture through signaling and physicochemical interactions and may also support selected metabolic processes. We use the term “mineral storm” as a hypothesis-generating description of this localized ionic flux, not as an established clinical mechanism.

#### 4.2.1 Ions as signaling molecules: architects of biofilm matrix and virulence

Elevated Ca^2+^ and P_i_ can act as environmental signals that influence bacterial gene expression and biofilm architecture. In *S. aureus* and *P. aeruginosa*, extracellular Ca^2+^ can activate sensing pathways associated with EPS production (Soe et al., 2021; Kolodkin-Gal et al., 2023). The magnitude and clinical relevance of these effects are likely to depend on pathogen, concentration, matrix composition, and local flow conditions.

Beyond transcriptional regulation, divalent cations like Ca^2+^ and, to a lesser extent, Mg^2+^, play a direct physicochemical role in matrix assembly. They act as ionic cross-linkers, bridging the anionic functional groups abundantly present within the biofilm’s EPS, such as carboxylates in uronic acids and P_i_ in eDNA or teichoic acids (Wang et al., 2019). This cross-linking dramatically increases the structural cohesion, viscoelasticity, and physical integrity of the biofilm. Phosphate ions also contribute to this signaling nexus; fluctuations in P_i_ concentration can serve as an environmental signal for nutrient stress or abundance, modulating global regulators that influence biofilm development, stress response, and the transition to a more persistent state (Li et al., 2024). Thus, the ionic milieu derived from bone dissolution actively scripts the architectural and defensive blueprint of the developing biofilm.

#### 4.2.2 Ions as essential nutrients: fueling the biofilm community

The mineral storm provides more than just signals; it delivers a concentrated broth of essential macro- and micronutrients that are often limiting in the otherwise nutrient-poor environment surrounding an inert implant. P_i_ is a prime example, being an indispensable component of phospholipids for cell membranes, the backbone of nucleic acids, and central energy currency molecules like adenosine triphosphate (ATP) (Lacerda-Abreu et al., 2020). The osteolytic release of P_i_ effectively fertilizes the biofilm, alleviating a critical nutritional limitation that would otherwise constrain bacterial density and metabolic activity (Neznansky et al., 2014). This abundance supports high rates of proliferation, underpinning the rapid expansion of the bacterial community within the protected biofilm niche and enabling the sustained production of virulence factors and matrix components.

Liberated Ca^2+^ and Mg^2+^ can serve as enzymatic cofactors, whereas P_i_ supports nucleic-acid, membrane, and energy metabolism (Neznansky et al., 2014; Pilchova et al., 2017; Lacerda-Abreu et al., 2020; Gu et al., 2025). These observations make it plausible that bone-derived ions influence bacterial physiology, but they do not establish that osteolysis alone supplies a growth-limiting nutrient pool in human implant infection. The proposed link between matrix dissolution and sustained biofilm growth should therefore be tested directly rather than treated as a proven feed-forward mechanism.

### 4.3 Degraded matrix components: collagen fragments, growth factors, and nanoparticles as active architects of the infectious niche

The relentless assault of biofilm-associated bacteria and osteoclasts on bone does not merely create structural damage. It generates a complex soup of degradation products (Dong et al., 2024). This material, derived from the fragmentation of both the organic collagen matrix and the inorganic mineral phase, is far from inert. Collagen peptides, embedded growth factors, and calcium phosphate nanoparticles become potent biological and physical modulators, actively reshaping the local microenvironment at the implant tissue interface (Pan et al., 2025). Their release establishes a self-amplifying feedback loop. Tissue destruction provides raw materials that enhance biofilm resilience, nutrient acquisition, and local immune dysregulation, cementing the chronicity of infection.

#### 4.3.1 Provision of alternative carbon and nitrogen sources, fundamentally shifting the local nutritional ecology

The enzymatic cleavage of type I collagen, orchestrated by both bacterial proteases like *S. aureus* aureolysin and V8 protease and host-derived matrix metalloproteinases (MMPs), floods the local milieu with oligopeptides and free amino acids. For bacteria, this represents a critical nutritional windfall (Zdzalik et al., 2012). Pathogens like *S. aureus* are adept nutrient scavengers, equipped with sophisticated transport systems such as the oligopeptide permease (Opp) complex and specific amino acid transporters (Teoh et al., 2021). The liberation of proline- and hydroxyproline-rich collagen fragments provides a high-quality nitrogen source, potentially supplanting other metabolic pathways. This access to host-derived peptides can support bacterial proliferation and survival deep within the biofilm, where diffusion of exogenous nutrients is limited. Furthermore, this metabolic shift can have profound downstream effects on virulence (Lensmire et al., 2020). Altered nutrient availability can influence global regulatory systems like accessory gene regulator (agr), potentially modulating toxin production and biofilm dynamics, thereby adapting bacterial behavior to the specific, nutrient-rich yet hostile environment of decaying bone.

Beyond collagen, the solubilization of the bone matrix also releases a cache of sequestered signaling molecules, most notably bone morphogenetic proteins (BMPs) and transforming growth factor-β (TGF-β) (Chen et al., 2026). While their primary role is in osteogenesis and immune modulation, their dysregulated release in an infectious context creates a paradoxical situation. These growth factors contribute to the chaotic and excessive bone remodeling seen in osteomyelitis, characterized by simultaneous, futile attempts at bone formation alongside rampant resorption (Siddiqui and Partridge, 2016). Whether this dysregulated signaling directly benefits biofilm persistence remains speculative; it may indirectly support the biofilm by sustaining metabolically active osteoblasts and stromal cells that bacteria can invade, or by creating a localized inflammatory milieu that diverts host immune resources, though direct evidence in orthopedic implant infection is limited.

#### 4.3.2 Integration into the biofilm scaffold, altering its physicochemical and structural properties

The degradation products do not merely dissolve into the fluid phase; many are physically incorporated into the evolving architecture of the biofilm itself. Collagen peptides and other bone-derived proteins can adsorb to bacterial surfaces and intertwine with the existing EPS. This proteinaceous integration may serve multiple functions: it can act as an additional ligand for adhesion to the implant or newly exposed bone surfaces, strengthen the mechanical cohesion of the biofilm, and provide a protective coating that masks pathogen-associated molecular patterns (PAMPs), potentially evading immune recognition (Pechaud et al., 2024).

Mineral dissolution may also alter the material properties of the biofilm matrix. Ca^2+^ and P_i_ can interact with anionic EPS components and, in some experimental systems, promote cross-linking or mineral deposition (Li et al., 2016; Pechaud et al., 2024; Wang et al., 2025). Such changes may increase matrix rigidity and reduce susceptibility to mechanical disruption. Their effect on antimicrobial transport is agent specific: mineralized or highly charged matrices can retard selected cationic agents, whereas many conventional antibiotics penetrate staphylococcal biofilms adequately (Darouiche et al., 1994; Tseng et al., 2013; Jacobs et al., 2022). Accordingly, physiological tolerance of embedded bacteria remains the dominant explanation for treatment failure in many settings, and the clinical contribution of biofilm biomineralization requires further validation.

These matrix effects should therefore be interpreted as one component of biofilm tolerance rather than as a universal diffusion barrier (Lebre et al., 2017; Idrees et al., 2021).

Thus, the bone matrix is not merely a passive substrate for biofilm attachment. It is an active participant in the infection, supplying both nutritional currency and literal building blocks, organic and inorganic, that fortify the biofilm community. This bidirectional exchange creates a uniquely resilient parasitic relationship: the biofilm dismantles tissue to construct its own fortified citadel, rendering the infection extraordinarily difficult to eradicate (Nguyen Ngoc et al., 2025). The bidirectional nature of this crosstalk is also summarized in **Table 1**.

## 5 Biofilm resilience and remodeling: navigating and reshaping a hostile host niche

### 5.1 Biofilm phenotypic plasticity and the evolution of antibiotic tolerance

#### 5.1.1 Persister cell formation: metabolic quiescence as a core survival strategy

A cornerstone of biofilm intransigence in implant-associated orthopedic infections is the formation of persister cells (Balaban et al., 2004). These constitute a metabolically dormant subpopulation that survives high-dose antibiotic exposure without acquiring genetic resistance. This phenotypic switch represents a reversible bet-hedging strategy at the population level, distinct from stable genetic mutations. Three related but distinct dormancy phenotypes should be clarified (Proctor et al., 2006).

Persister cells are subpopulations in reversible metabolic quiescence that survive antibiotic exposure without genetic change. Small-colony variants (SCVs) are slow-growing, nutritionally deficient subpopulations often associated with intracellular persistence. Viable but non-culturable (VBNC) cells represent a deeper dormancy state characterized by profound metabolic shutdown and loss of culturability under standard laboratory conditions.

Within the dynamic microenvironment of an infected bone-implant interface, stress abounds. Nutrient limitation, immune cell attack, and fluctuating oxygen levels create relentless pressure. In response, a fraction of the biofilm community enters deep metabolic quiescence.

Recent single-cell analyses in *Escherichia coli* (*E. coli*) have demonstrated that bacterial dormancy exists along a continuum of metabolic depth, correlating with intracellular ATP depletion and protein aggregation (Pu et al., 2019). While direct comparative studies of persister, SCV, and VBNC phenotypes within a unified continuum remain limited, the concept of graded dormancy provides a useful heuristic for understanding the spectrum of bacterial survival strategies in chronic orthopedic infections. Persister cells occupy a shallower dormancy state from which resuscitation remains readily reversible, whereas VBNC cells reside in deeper dormancy that may require specific stimuli for recovery or may be irreversible. The quantity of protein aggregates and the cell’s capacity to recruit ATP-dependent disaggregation chaperones such as the DnaK-ClpB system determine resuscitation lag time.

The clinical consequence of persister cell formation is profound. Following cessation of therapy, these dormant cells resume metabolic activity and reseed the biofilm, leading to relapse (Lu et al., 2022). In orthopedic implant infections, this phenomenon is particularly concerning given the unique bone microenvironment. Bone marrow stem cell niches are immune-privileged sites containing regulatory T cells and immature myeloid-derived suppressor cells (MDSCs), creating a microenvironment conducive to pathogen survival (Tuttobene et al., 2025). Various dormant bacteria can infect mesenchymal and hematopoietic stem cells, using these cells as long-term reservoirs. Infected stem cells transmit pathogens to their progeny, facilitating traffic to joints and entheses. Local inflammatory cytokines such as tumor necrosis factor may induce bacterial metabolic shutdown, yet certain pathogens exploit these same signals to promote an immunosuppressive milieu that favors bacterial survival (Sahukhal et al., 2020).

Persister cell formation in orthopedic biofilm infections is therefore dual-driven. It involves both an intrinsic bacterial response to nutrient and antibiotic stress, and an adaptive response to the host bone marrow and inflammatory microenvironment. This bidirectional crosstalk secures a protected, dormant niche and underscores a therapeutic imperative: strategies must either prevent entry into deep dormancy or target the resuscitation mechanisms critical for relapse.

The duration of persister survival in periprosthetic tissue remains uncertain. Persisters are phenotypically tolerant, and simply prolonging conventional antimicrobial therapy is unlikely to eradicate them (Arvieux and Common, 2019). Surgical debridement and removal or exchange of infected hardware remain essential when indicated. Metabolic reactivation has shown promise only in preclinical models; no clinically established adjuvant currently replaces surgical source control.

#### 5.1.2 Fortification of physicochemical barriers: a synergistic, multi-mechanism defense

Beyond entering a metabolically dormant state, biofilm communities mount a robust, active defense. They reinforce their physicochemical barriers. A fortified enclave emerges, recalcitrant to both host immune clearance and antimicrobial penetration. This defensive fortification is not a singular event. It is a synergistic orchestration of matrix remodeling, altered porosity, and strategic molecular deployment.

A key architectural strategy involves reducing matrix porosity. This physically excludes immune effectors. In *S. aureus*, extracellular adherence proteins (Eaps) are crucial in this process. EapH1 and EapH2 decrease biofilm porosity. Their impact is particularly pronounced for molecules in the 70–150 kDa range. Macrophage infiltration is thereby limited. So too is the entry of other soluble antimicrobial factors. This dense, less porous architecture transforms the biofilm. It is no longer a mere aggregate. It becomes a shielded microenvironment (Bhattacharya et al., 2025).

The matrix is further reinforced by exopolysaccharides, eDNA, proteins, and teichoic acids. In staphylococci, deacetylation of poly-N-acetylglucosamine by IcaB promotes surface retention and resistance to cationic antimicrobial peptides (Nguyen et al., 2020). Foreign-body infection also impairs local neutrophil function. Classic tissue-cage experiments showed that neutrophils interacting with an implant undergo partial degranulation, reduced superoxide production, and diminished bactericidal activity against *S. aureus* (Zimmerli et al., 1984). This phenomenon, rather than T-cell failure, directly compromises bacterial clearance at the implant surface.

Biofilms actively manipulate this barrier in response to environmental cues. Mechanical shear stress can upregulate polysaccharide expression. Matrix robustness is thereby tailored to the specific hydrodynamic conditions of the implant site. The cumulative effect of these adaptive mechanisms is profound. Porosity control, charge modification, and composite matrix assembly establish a dynamic, multi-layered defense. This barrier does not merely passively withstand host pressures. It actively shapes a hostile perimeter. This perimeter defines the BBI (Hou et al., 2018; Lawal et al., 2021). Bacterial persistence within the osseous niche is thereby ensured.

### 5.2 Metabolic flexibility: commensalism and cross-feeding on bone-derived nutrients

The bone-implant interface represents a uniquely hostile environment, characterized by nutrient scarcity and a fierce immunological landscape. For colonizing bacteria, survival here is not passive. They must actively overcome these compounding pressures (Dong et al., 2024). Biofilm communities display a remarkable degree of metabolic plasticity. Their strategy extends beyond simple resource scavenging; instead, they construct sophisticated cooperative networks to exploit the distinct nutritional profile of bone tissue.

The bone matrix itself functions as a primary nutrient reservoir (Garcia-Moreno et al., 2022; Zhan et al., 2025). A process of osteolysis releases a complex mixture of organic and inorganic compounds. This degradation can be driven by host osteoclasts or directly instigated by bacterial factors (Yoshimoto et al., 2022; Dong et al., 2024). The resulting liberation of collagen peptides, hydroxyproline, Ca^2+^, and P_i_ provides a vital influx of resources (Zhan et al., 2025). Pioneer microorganisms initiate this process by secreting a suite of extracellular enzymes. Proteases, nucleases, and lipases degrade structural bone matrix proteins, while eDNA and cellular debris are similarly broken down (Bhattacharya et al., 2025; Hasan and Aggarwal, 2025). Through this metabolic hijacking, what was once a structural scaffold of the host is transformed into a self-renewing nutrient supply system. This fuels biofilm expansion in a microenvironment otherwise devoid of readily available nutrients (Li et al., 2025).

This metabolic strategy is significantly amplified in polymicrobial consortia. Such communities are characteristic of chronic implant-associated orthopedic infections. Interspecies metabolic cross-feeding emerges as a cornerstone of community resilience. It also enhances pathogenicity. Different species partition metabolic labor. Interdependent syntrophic relationships are created. Overall fitness is enhanced (Moriarty et al., 2022; Dong et al., 2024). One example illustrates this cooperation. *Enterococcus faecalis* (*E. faecalis*) and *E. coli* interact under iron limitation. This is a key component of host nutritional immunity. *E. faecalis* is remarkably tolerant to iron starvation. It constitutively exports L-ornithine via the arginine deiminase pathway D (ArcD) antiporter. The exported metabolite serves as a critical cue for *E. coli*. It stimulates biosynthesis of the siderophore enterobactin. This molecule chelates and imports otherwise inaccessible host iron. Metabolic facilitation enables *E. coli* to thrive. Robust biofilm formation follows (Cui et al., 2026). Conditions that would normally restrict growth are thereby overcome.

Such interactions extend beyond iron acquisition. Facultative anaerobes like *Staphylococcus* spp. may consume local oxygen. Hypoxic niches are created. These favor the proliferation of obligate anaerobes. *Cutibacterium acnes* and *Finegoldia magna* benefit. In return, these anaerobes ferment amino acids or peptides. Short-chain fatty acids and other metabolites are produced. These serve as nutrients for neighboring species (Dong et al., 2024). This mutualistic metabolic exchange forges resilient, interdependent communities. These exhibit markedly enhanced antibiotic resistance. They are better equipped to evade host immune clearance compared to single-species biofilms (Moriarty et al., 2022; Dong et al., 2024).

The metabolic adaptability of these biofilms extends even further. They can scavenge nutrients from byproducts of host inflammation. Infiltrating neutrophils and macrophages release substantial amounts of lactate during the inflammatory response (Dong et al., 2024). Certain bacteria, such as *P. aeruginosa*, readily utilize this molecule as a preferred carbon source (Dong et al., 2024; Li et al., 2025). Consequently, the very immune response intended to control the infection can inadvertently fuel its chronicity.

Through this coordinated interplay of multiple strategies, the biofilm establishes a self-sustaining metabolic ecosystem. It directly harvests nutrients from bone. It forges complex inter-species metabolic interactions. This establishes a persistent infectious cycle at the implant site. This cycle is exceptionally difficult to disrupt.

### 5.3 Active niche engineering: secreted effectors exacerbate osteolysis and microenvironment deterioration

Biofilms do more than simply adapt. They actively reshape their surroundings through a sophisticated arsenal of secreted virulence factors. The immediate environment undergoes catastrophic remodeling. This aggressive reshaping directly fuels bone destruction. Simultaneously, an immunosuppressive and biofilm-friendly milieu is created (Masters et al., 2022; Dong et al., 2024).

The biofilm matrix itself forms a formidable physical and chemical shield. Polysaccharides, eDNA and proteins compose this structure (Jakubovics et al., 2021). Biofilms produce factors like poly-γ-glutamic acid in *Bacillus* species. Staphylococci generate PIA. These chelate cationic antimicrobial peptides. Phagocyte chemotaxis is inhibited. Reactive oxygen and nitrogen species are neutralized (Cardoso et al., 2021; da Cruz Nizer et al., 2024). This matrix acts as a sink for key immune attack mechanisms. The host’s frontline defenders are effectively disarmed (Evans et al., 2025).

Chronic inflammatory stimuli drive pathological tissue remodeling. Necrotic debris, acidic metabolites, and expanding hypoxic zones accumulate. Persistent bacterial stimuli fuel this process. The dysregulated environment damages local bone-forming cells. Osteoblasts and mesenchymal stem cells are functionally compromised (Zhang et al., 2025). Any reparative response is thereby aborted. Fibroblast proliferation and collagen deposition are promoted instead. An avascular, fibrotic capsule forms around the infection site. This capsule functionally isolates the biofilm. Systemic circulation and immune surveillance are blocked. Antibiotics are rendered ineffective. A state of chronic, intractable infection is solidified (Zhang et al., 2025).

Thus, through concerted niche engineering, biofilms actively construct a protected, osteolytic microenvironment. This niche constitutes the fundamental pathological hallmark. It also represents the central therapeutic challenge in orthopedic implant infections. The specific cellular mechanisms underlying bone cell hijacking are examined in the following section.

## 6 Active subversion of bone homeostasis and immune surveillance

### 6.1 Modulating the bone remodeling program

In healthy bone, osteoclasts are multinucleated cells derived from the monocyte-macrophage lineage. In response to macrophage colony-stimulating factor and RANKL, they resorb mineralized bone and couple this activity to osteoblast-mediated formation, thereby supporting skeletal renewal and mineral homeostasis (Siddiqui and Partridge, 2016; Tsukasaki and Takayanagi, 2019). In infection and other inflammatory bone disorders, persistent RANKL, TNF-α, IL-1β, and pattern-recognition signaling can uncouple resorption from formation, producing excessive or prolonged osteoclast activity rather than a novel osteoclast function. Biofilm-associated bacteria, particularly S. aureus, exploit this dysregulated remodeling program and also impair osteoblasts and osteocytes, shifting the cellular triad toward net bone loss.

#### 6.1.1 Pathological activation of osteoclasts: molecular mimicry and inflammatory cytokine storm

Biofilm-associated bacteria directly target the cellular machinery of bone resorption. *S. aureus* exemplifies this strategy. The pathogen secretes toxins and adhesins that stimulate osteoclastogenesis through multiple pathways (Widaa et al., 2012; Mouton et al., 2021). Protein A, a key surface virulence factor, binds the Fc region of IgG to form bacterial immune complexes (Ren et al., 2017). These complexes engage activating Fcγ receptors (FcγRs) on osteoclast precursors, namely FcγRIII and FcγRIV. Intracellular signaling is triggered via Syk kinase. This FcγR-mediated signal synergizes with concurrent activation of Toll-like receptor 2 (TLR2). Lipoproteins and other staphylococcal components drive this activation. A robust, myeloid differentiation primary response 88 (MyD88)-dependent activation of nuclear factor-κB (NF-κB) and nuclear factor of activated T cells cytoplasmic 1 (NFATc1) follows. The latter serves as master regulator of osteoclast differentiation (Negishi-Koga et al., 2015). This FcγR-TLR2 crosstalk drives excessive osteoclast formation. It also amplifies production of potent pro-osteoclastogenic cytokines. TNF-α and interleukin-1β (IL-1β) are upregulated by the precursors themselves (Yao et al., 2024). A self-amplifying inflammatory feed-forward loop is created.

The pivotal roles of Protein A in osteoclast activation and of SarA in regulating biofilm virulence have been extensively characterized by independent research groups, including those led by Kielian and Smeltzer, providing a mechanistic foundation for understanding *S. aureus*-driven bone destruction in implant infection models (Beenken et al., 2003; Widaa et al., 2012; Kamohara et al., 2020).

Bacterial products further modulate the RANKL/OPG axis. This system serves as master regulator of bone remodeling (Tsukasaki and Takayanagi, 2019). *S. aureus* infection induces osteoblasts and activated T cells to overexpress RANKL (Wang et al., 2018; Campbell et al., 2024). Sustained overproduction of this cytokine, coupled with decreased expression of its decoy receptor OPG, creates a potent and persistent osteoclastogenic signal. This imbalance is further exacerbated by pro-inflammatory cytokines. TNF-α and interleukin-6 (IL-6) are abundantly produced. Immune cells and infected bone cells within the biofilm microenvironment serve as sources. These molecules synergistically enhance RANK and RANKL signaling. Osteoclast precursor differentiation is accelerated (Luo et al., 2018; Yin et al., 2025).

Polymicrobial communities may amplify destructive signaling by presenting multiple pathogen-associated molecular patterns concurrently. Peptidoglycan-derived signals engage TLR2 and NOD pathways, whereas lipopolysaccharide engages TLR4; convergence on TRAF6 and NF-κB can increase pro-osteoclastogenic cytokine production and osteoclast differentiation in preclinical inflammatory-bone-loss models (Aoki et al., 2023; Huang et al., 2023). Because these data derive from experimental systems rather than direct comparisons in human implant infection, the magnitude of such synergy at the clinical BBI remains uncertain.

Beyond this cytokine storm, *S. aureus* establishes an even more intimate relationship with osteoclasts. Remarkably, osteoclasts can serve as intracellular reservoirs for the bacterium. This contrasts sharply with their progenitor cells. Bone marrow macrophages typically kill internalized *S. aureus*. Osteoclasts differentiated via RANKL signaling behave differently. They support robust intracellular bacterial replication. This permissive niche depends on RANKL-induced activation of specific pathways. The transcription factor NFATc1 is activated. The alternative NF-κB pathway is similarly engaged (Krauss et al., 2019). Consequently, osteoclasts are doubly compromised. They are hyper-activated to resorb bone. Simultaneously, they are exploited to protect and propagate the pathogen. Bone destruction is thereby directly linked to bacterial survival. Direct evidence that osteoclasts serve as replication permissive reservoirs derives from murine models, and the quantitative contribution of this niche to human relapse remains unestablished; we therefore present this mechanism as a plausible and testable component of the framework rather than as a proven clinical driver (Krauss et al., 2019).

#### 6.1.2 Suppression of osteoblast function: pro-apoptotic signaling and differentiation blockade

Biofilms compromise osteoblasts through multiple avenues. Repair mechanisms collapse. A favored tactic is outright cell killing. Protein A from *S. aureus* engages tumor necrosis factor receptor 1 (TNFR1). Death cascades ignite (Hu et al., 2022). Pore-forming toxins, phenol-soluble modulins among them, rupture membranes. Necrosis follows. Infected osteoblasts begin secreting tumor necrosis factor-related apoptosis-inducing ligand (TRAIL). This ligand, related to TNF, eliminates both infected neighbors and innocent bystanders (Mendoza Bertelli et al., 2016), losses mount.

The harm extends beyond cytotoxicity. *S. aureus* invades osteoblasts through fibronectin-binding protein (FnBP) and integrin α5β1 (Crepin et al., 2026), suppressing the osteogenic program through reduced Runt-related transcription factor 2 (RUNX2), collagen type I alpha 1 chain (COL1A1), alkaline phosphatase, and matrix mineralization. Post-transcriptional and epigenetic changes, including loss of pro-osteogenic microRNA-24 signaling, reinforce this inhibition (Josse et al., 2015). The resulting imbalance suppresses osteoblast-mediated bone formation while osteoclast-mediated resorption remains excessive, producing pathological uncoupling of bone remodeling.

#### 6.1.3 Disruption of the osteocyte network: interfering with mechano-chemical coupling

Osteocytes may provide a protected intracellular niche for persistent *S. aureus*. The organism can adopt a slow-growing small-colony-variant phenotype associated with reduced cytotoxicity and prolonged intracellular survival (Yang et al., 2018; Gunn et al., 2024). Because osteocytes are abundant and long lived, this reservoir is biologically plausible; however, its quantitative contribution to relapse in human implant infection remains uncertain.

Once infected, osteocytes become active agents of bone destruction. They also impair healing. Expression of pro-osteoclastogenic factors is upregulated. RANKL is prominently among these (Yoshimoto et al., 2022). Matrix-degrading enzymes are secreted. Cathepsin K and MMPs serve this function. Periprosthetic osteolysis directly contributed to (Qin et al., 2024). Crucially, *S. aureus* infection in osteocytes stimulates production of sclerostin (Sost). This is the product of the *Sost* gene, which is a potent inhibitor of the canonical wingless-type (Wnt) and β-catenin signaling pathway. This pathway serves as critical driver of osteoblast differentiation and bone formation. By corrupting osteocyte signaling in this way, biofilms promote resorption. They also systemically suppress the bone’s anabolic response (Jiao et al., 2023). The normal bone remodeling cycle is effectively decoupled and disabled.

### 6.2 Polymicrobial consortia and the vicious cycle

Most mechanistic models of the BBI derive from monomicrobial *S. aureus* systems. Polymicrobial infection is nevertheless common in open-fracture settings and can alter the interface through metabolic cooperation, combined immune stimulation, and community-level persistence. These interactions may change both the rate and the structure of the proposed cycle.

First, metabolic interdependence strengthens the nutrient arm of the cycle. Interspecies cross-feeding, such as *E. faecalis* supplying ornithine to support *E. coli* siderophore production under iron limitation, and the creation of hypoxic microniches by facultative anaerobes that favor obligate anaerobes such as Cutibacterium acnes, builds syntrophic networks that sustain community growth under nutritional immunity (Dong et al., 2024; Li et al., 2025). Biofilms embedded in such networks exhibit enhanced antibiotic tolerance and immune evasion compared with single species counterparts, accelerating Phase 3 fortification.

Second, synergistic immune and bone cell modulation amplifies Phase 1 osteolysis. Peptidoglycan acting through TLR2 and NOD receptors cooperates with lipopolysaccharide acting through TLR4 to induce significantly greater osteoclast formation and bone resorption than either component alone, via enhanced TRAF6 and NF κB signaling (Aoki et al., 2023; Huang et al., 2023). Polymicrobial PAMP exposure thus steepens the bone resorption gradient that feeds the mineral storm, potentially converting a slow smoldering cycle into a rapidly escalating one.

Third, community level persistence frustrates treatment of Phase 4. Therapeutic strategies that eliminate only the dominant pathogen, a “kill the leader” approach, have produced short-term improvement followed by relapse in polymicrobial bone graft biofilms, because residual species exploit the liberated niche and rebuild the consortium (Cardoso et al., 2021; Jakubovics et al., 2021; da Cruz Nizer et al., 2024; Evans et al., 2025; Zhang et al., 2025; Zhang et al., 2025). Consequently, the vicious cycle in polymicrobial BBI is not merely a sum of species specific cycles but a qualitatively distinct, self righting system that demands comprehensive community coverage rather than single pathogen targeting. This dimension is revisited in the Limitations.

### 6.3 Temporal dynamics of the BBI crosstalk and critical windows for intervention

BBI crosstalk is unlikely to remain constant over time. During the earliest phase after contamination, bacterial attachment may still be reversible, organisms are metabolically active, and intracellular reservoirs and mature matrix defenses have not yet formed. This period is therefore expected to be most responsive to debridement, appropriate antimicrobial therapy, and, when clinically suitable, implant-retention strategies (Tschudin-Sutter et al., 2016).

As infection progresses, irreversible adhesion, EPS accumulation, intracellular invasion, altered bone remodeling, and local immune dysfunction may progressively reduce therapeutic accessibility (Heim et al., 2015a; Heim et al., 2015b; Scherr et al., 2015). Clinical outcomes are consistent with this concept: implant retention is more successful in early than in late infection, even though pathogen identity does not shift systematically with time (Kuehl et al., 2019; Corrigan et al., 2022). The proposed phases should not be treated as fixed chronological categories; they are overlapping, testable states whose timing will vary with pathogen, host, and implant factors.

### 6.4 The implant surface and material as active components of the BBI

The implant is conventionally treated as an inert scaffold, but its surface is an active component of the BBI. It is not literally immunoprivileged; rather, an avascular abiotic surface cannot itself recruit immune cells or deliver systemic antibiotics, and it is rapidly covered by a host-protein conditioning film that can support both bacterial attachment and host-cell integration. Surface topography, roughness, free energy, wettability, and charge shape that conditioning film and influence the initial competition between bacteria and host cells for the surface (Rather et al., 2021; Lu et al., 2022; Pietrocola et al., 2022). Fibrous encapsulation and local phagocyte dysfunction can subsequently create a relatively immune-protected compartment without eliminating immune activity (Zimmerli et al., 1984; Scherr et al., 2015; Schoberleitner et al., 2024).

Material class modifies, but does not independently determine, infection risk. Titanium and titanium alloys promote osseointegration, yet rough or porous regions provide more attachment area; stainless steel and cobalt-chromium alloys are mechanically useful but can release corrosion products or wear particles; and polymers such as polymethylmethacrylate, ultra-high-molecular-weight polyethylene, and polyetheretherketone can support protein adsorption and biofilm while generating inflammatory debris. Dense ceramics generally produce less wear but are not resistant to conditioning-film formation or microbial colonization. Wear particles and metal ions can activate macrophages, promote RANKL-dependent osteoclastogenesis, and impair osteoblast viability and differentiation, thereby linking material degradation to delayed healing and osteolysis (Piuzzi et al., 2024; Yao et al., 2025; Yi et al., 2025). Importantly, bacterial affinity cannot be ranked by material name alone. *In vitro* comparisons of PEEK and titanium show that adhesion varies with surface finish, protein conditioning, bacterial species, and sampling time; rougher machined PEEK supported greater staphylococcal adhesion in one model, whereas species-dependent patterns were observed in spinal-cage comparisons (Rochford et al., 2014; Krätzig et al., 2021).

### 6.5 Reprogramming the local immune microenvironment

To complement the disruption of bone integrity, biofilms orchestrate a profound reprogramming of the local immune response, transforming it from a defensive force into a facilitator of chronicity.

Neutrophil granulocytes are the primary executors of bacterial killing at the BBI through phagocytosis, degranulation, and oxidative burst, with macrophages contributing additional clearance. T cells act mainly as regulators of these innate responses. Suppression of T-cell activity may therefore weaken bacterial control indirectly, but it should not be described as the principal defect in direct bacterial killing (Heim et al., 2014; Horn et al., 2024).

#### 6.5.1 Induction of macrophage M2-like polarization: establishing immune tolerance

A central pillar of biofilm-mediated immune evasion is the manipulation of macrophage plasticity (Brandquist and Kielian, 2025). The initial M1-like pro-inflammatory response is actively subverted. This shift is necessary yet ultimately undermined. *S. aureus* biofilms drive polarization toward an anti-inflammatory, pro-reparative M2-like phenotype (Seebach et al., 2023). It is now recognized that macrophage polarization in chronic infection exists along a continuum rather than as a binary M1/M2 dichotomy; however, for clarity, we refer to the M2-like phenotype to describe the anti-inflammatory, pro-reparative end of this spectrum observed in *S. aureus* biofilm contexts chronic exposure to biofilm antigens mediates this transition. An altered metabolic microenvironment contributes similarly. Biofilm structures can sequester nutrients (Bertrand et al., 2024). Macrophages are thereby forced to rely on oxidative phosphorylation. This metabolic profile is associated with anti-inflammatory polarization.

This phenotypic shift has concerning consequences for infection control. M2-like macrophages exhibit diminished capacity for phagocytosis. Bacterial killing is similarly impaired (Scherr et al., 2015). Production of pro-inflammatory cytokines is reduced. Interleukin-12 (IL-12) and TNF-α fall into this category (Yan et al., 2025). Concurrently, secretion of immunosuppressive mediators increases. Interleukin-10 (IL-10) serves as a prime example (Seebach et al., 2023). The primary role of these cells switches fundamentally. Pathogen elimination is abandoned. Tissue repair and inflammation resolution take precedence. These functions prove maladaptive when a biofilm persists. Thus, the biofilm co-opts the macrophage response (Yu et al., 2026). A state of local immune tolerance is established. The host’s defenses are actively suppressed rather than engaged. The mechanisms underlying biofilm-induced macrophage dysfunction in orthopedic infection have been extensively investigated by Kielian and colleagues. Their work demonstrated that *S. aureus* biofilms induce macrophage dysfunction through leukocidin AB and alpha-toxin (Scherr et al., 2015), and that IL-10 production by MDSCs contributes to bacterial persistence during orthopedic biofilm infection (Heim et al., 2015a; Heim et al., 2015b). These studies provide critical evidence linking specific bacterial virulence factors to immune cell reprogramming in the bone microenvironment.

#### 6.5.2 Promotion of fibrotic encapsulation: constructing a physical and immune barrier

The dysregulated immune response to a chronic biofilm often culminates in a specific structural outcome. A dense fibrotic capsule forms around the implant (Schoberleitner et al., 2024). This process is largely driven by sustained activity of anti-inflammatory macrophages. Other cells contribute similarly. They secrete pro-fibrotic factors. TGF-β serves as a key example (Eom et al., 2025). Fibrosis represents a host attempt to isolate the infection. It ultimately serves the biofilm’s interests instead.

The resulting scar tissue is avascular and collagen-rich. It acts as a formidable physical barrier. Diffusion of oxygen and nutrients is severely impeded. More critically, penetration of circulating immune cells is drastically reduced. Systemically administered antimicrobial agents face the same obstruction. This encapsulation creates a protected niche. The biofilm is effectively walled off from immune surveillance (Liu et al., 2026; Zhang et al., 2026). Pharmacological intervention is similarly excluded. Mechanical debridement often becomes a necessity for treatment (Yi et al., 2025). The high failure rates of antibiotic therapy alone are directly contributed to by this structural isolation.

#### 6.5.3 Recruitment and activation of MDSCs: deepening immunosuppression

Emerging evidence underscores a specific cellular mechanism. MDSCs reinforce the immunosuppressed niche around biofilms. These heterogeneous populations of immature myeloid cells are powerful suppressors. Both innate and adaptive immunity fall under their control. The chronic inflammatory milieu serves as potent chemoattractant. Specific factors released from the biofilm microenvironment contribute similarly (Heim et al., 2014; Horn et al., 2024).

Once recruited, MDSCs deploy an arsenal of suppressive mechanisms. Arginase-1 is expressed at high levels. Local L-arginine is thereby depleted. This amino acid is essential for T cell proliferation and function. Reactive oxygen and nitrogen species are also produced. These can induce T cell apoptosis. Natural killer (NK) cell activity is inhibited. The adaptive immune response is compromised. The principal consequence of T cell suppression in this setting is indirect: without cytokine licensing from functional T cells, the neutrophil granulocytes and macrophages that execute the actual bacterial killing at the interface operate below capacity. Local immunosuppression is deepened by their presence (Heim et al., 2014; Horn et al., 2024). Some immune recognition may occur. It cannot mount an effective effector response (Zhang et al., 2026). The chronicity of the infection is thereby cemented.

In summary, the biofilm’s strategy for survival is multifaceted. Bone cells are co-opted. Surrounding tissue architecture is dismantled. Simultaneously, the host immune system is reprogrammed. A state of dysfunction and tolerance ensues (Brandquist and Kielian, 2025). **Figure 2** provides a visual summary of this immune reprogramming. It depicts how biofilms manipulate macrophages, MDSCs, and T cells. An immunosuppressive microenvironment is established. The multifaceted reprogramming of the local immune microenvironment is also comprehensively summarized in **Table 2**. This bidirectional interaction targets structural integrity. Immune defense is similarly attacked. A self-reinforcing pathogenic niche is created. It is exceptionally resistant to host clearance. Therapeutic intervention is similarly thwarted (Zhang et al., 2026). This defines the fundamental challenge in treating orthopedic implant-associated infections.

**Figure 2:**
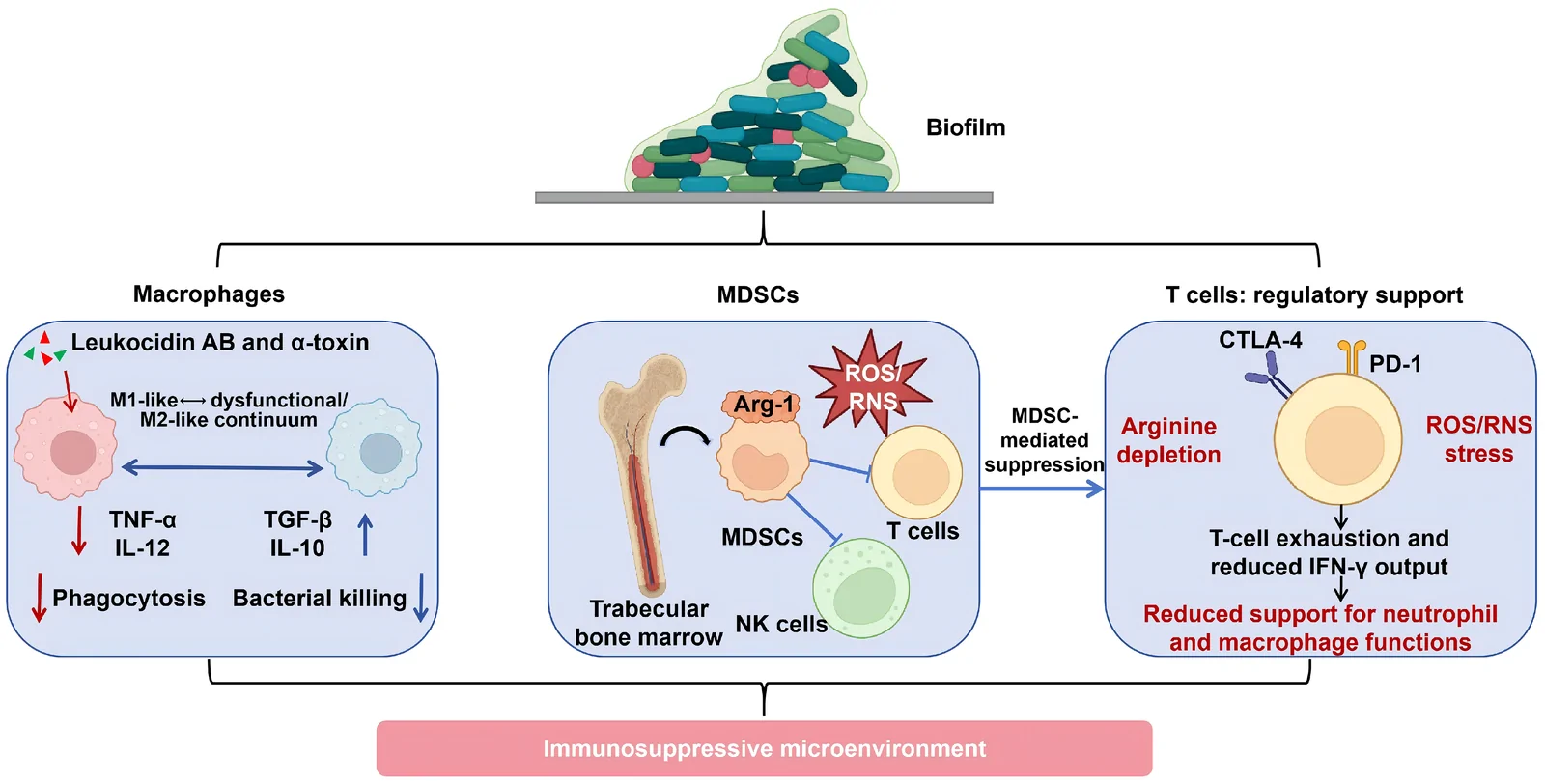
Biofilm-induced reprogramming of the local immune microenvironment. Biofilm-derived signals promote a shift along the macrophage continuum toward dysfunctional or M2-like states, with reduced TNF-α and IL-12 production, impaired phagocytosis and bacterial killing, and increased TGF-β and IL-10 signalling. Myeloid-derived suppressor cells inhibit T-cell and natural killer-cell activity through arginase-1-dependent arginine depletion and the production of reactive oxygen and nitrogen species. T-cell exhaustion, marked by PD-1 and CTLA-4 signalling and reduced IFN-γ output, diminishes regulatory support for neutrophil and macrophage function rather than directly impairing bacterial killing by T cells. Together, these changes establish an immunosuppressive microenvironment that favours biofilm persistence. Arg-1, arginase-1; CTLA-4, cytotoxic T-lymphocyte-associated protein 4; IFN-γ, interferon-γ; MDSCs, myeloid-derived suppressor cells; NK, natural killer; PD-1, programmed cell death protein 1; RNS, reactive nitrogen species; ROS, reactive oxygen species.

**Table 2:** Biofilm-induced immune cell dysfunction.

Cell type	Phenotypic shift	Functional changes	Consequence	References
Macrophages	Polarization from pro-inflammatory M1 to anti-inflammatory M2 phenotype; metabolic shift to oxidative phosphorylation	Phagocytosis capacity is reduced; bacterial killing is impaired; production of IL-12 and TNF-α decreases; secretion of IL-10 and TGF-β increases; leukocidin AB and alpha-toxin-mediated dysfunction.	Local immune tolerance develops; fibrotic encapsulation is promoted; biofilm persistence is facilitated.	(Scherr et al., 2015; Seebach et al., 2023; Bertrand et al., 2024; Brandquist and Kielian, 2025; Yan et al., 2025; Yu et al., 2026)
MDSCs	Expansion in bone marrow and recruitment to infection site; granulocytic subset predominance	Arginase-1 expression increases; reactive oxygen and nitrogen species are produced; T cell proliferation and function are inhibited; NK cell activity is suppressed.	Adaptive immune regulation is suppressed; antigen-specific responses are weakened; local persistence may be facilitated.	(Heim et al., 2014; Horn et al., 2024; Mitroulis et al., 2024)
T Cells	Regulatory dysfunction with PD-1 and CTLA-4 upregulation and altered glycolysis/HIF-1α signaling	Proliferative capacity diminishes; IFN-γ production and cytotoxicity are impaired; activation and cytokine support for phagocytes are impaired through arginine depletion and oxidative stress.	Loss of T cell licensing impairs neutrophil and macrophage activation; bacterial clearance, executed chiefly by neutrophil granulocytes, is compromised indirectly; minimal residual bacterial burden can reactivate infection.	(Zhang et al., 2026)

MDSCs, myeloid-derived suppressor cells; IL-12, interleukin-12; TNF-α, tumor necrosis factor-α; IL-10, interleukin-10; TGF-β, transforming growth factor-β; NK, natural killer; PD-1, programmed death-1; CTLA-4, cytotoxic T-lymphocyte-associated protein-4; HIF-1α, hypoxia-inducible factor-1α; IFN-γ, interferon-γ.

## 7 An integrative paradigm: the self-perpetuating “vicious cycle” at the BBI and its clinical implications

### 7.1 A self-amplifying cycle: from osteolysis to biofilm fortification and back

The core pathophysiology can be modeled as a four-phase, interlocking cycle. It perpetually operates at the BBI. This self-perpetuating vicious cycle is depicted in **Figure 3**.

**Figure 3:**
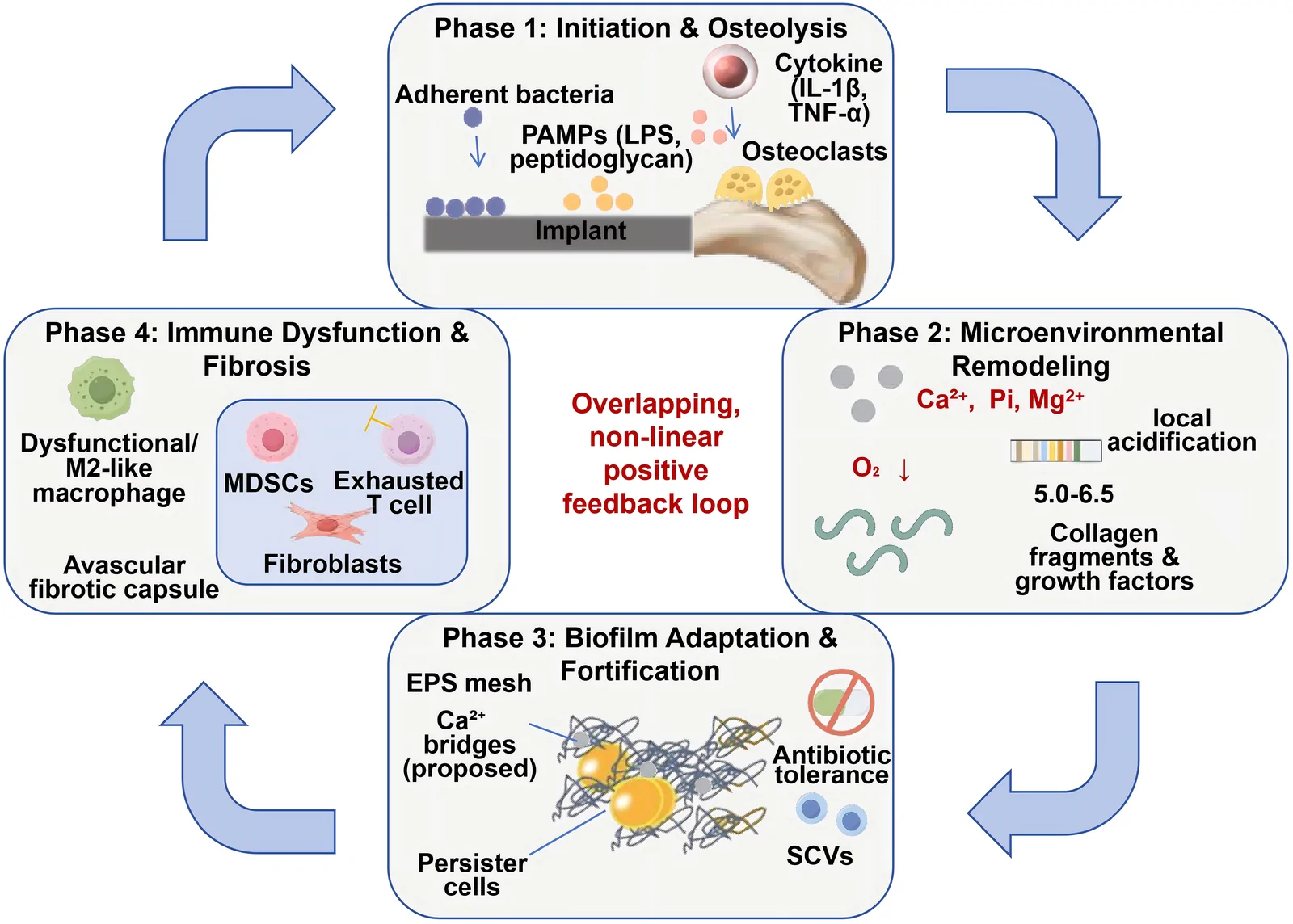
Overlapping phases of the self-reinforcing bone–biofilm interface cycle. The proposed cycle comprises four overlapping and non-linear phases. Phase 1 includes bacterial adhesion, inflammatory signalling and osteoclast-mediated bone resorption. Phase 2 comprises hypoxia, local acidification and the release of Ca^2+^, inorganic phosphate, Mg^2+^, collagen fragments and matrix-associated growth factors. Phase 3 involves extracellular polymeric substance remodelling, persister formation, small-colony variants and physiological antibiotic tolerance; Ca^2+^-mediated matrix reinforcement is presented as a proposed mechanism. Phase 4 includes dysfunctional or M2-like macrophages, myeloid-derived suppressor cells, T-cell exhaustion and avascular fibrotic encapsulation. These phases constitute a conceptual framework and should not be interpreted as a fixed chronological sequence in every patient. EPS, extracellular polymeric substance; MDSCs, myeloid-derived suppressor cells; PAMPs, pathogen-associated molecular patterns; P_i_, inorganic phosphate; SCVs, small-colony variants.

The proposed BBI cycle can be organized into four overlapping phases. Initial bacterial adhesion and innate inflammation promote phagocyte degranulation and RANKL-dependent osteoclastogenesis. Subsequent bone resorption and tissue injury alter pH, oxygen tension, ionic composition, and nutrient availability. These changes select for persistent phenotypes and may reinforce the EPS matrix. Chronic infection then sustains macrophage dysfunction, MDSC accumulation, impaired adaptive regulation, and fibrotic encapsulation (von Eiff et al., 1997; Loss et al., 2019; Niu et al., 2024; Almatroudi, 2025; Li and Yao, 2025). SCVs are associated particularly with prolonged subinhibitory antimicrobial exposure rather than being generated by biofilm growth alone (von Eiff et al., 1997; Loss et al., 2019). This four-phase scheme is an organizing heuristic, not a directly observed sequence in every patient.

This creates a positive feedback loop. Host executed osteolysis, sustained by biofilm driven inflammation, begets biofilm fortification. This in turn begets more profound osteolysis and immune evasion. Each revolution of the cycle deepens the pathology. The interface becomes increasingly hostile to therapeutic intervention.

### 7.2 Clinical translation: explaining chronicity, treatment failure, and recurrence

This integrative model provides a mechanistic framework that may illuminate the core clinical challenges in managing PJI and FRI. The model’s explanatory power lies in linking molecular and cellular events to patient-centered outcomes (Peterlin et al., 2026).

The root of chronicity and persistence becomes clear. The cycle explains why infections become self-sustaining ecosystems. The biofilm evolves from a passive colonizer into an integrated pathological entity. It actively maintains the conditions for its own survival (Almatroudi, 2025). Intracellular reservoirs within osteocytes and osteoclasts are safeguarded within this dysregulated niche. They function as a protected seed bank (Piuzzi et al., 2024). Bacterial persistence is ensured despite host defenses and intermittent antimicrobial pressure.

Antimicrobial failure is multifactorial. Restricted diffusion may contribute for selected agents, particularly some cationic antimicrobials, but penetration of many conventional antibiotics through staphylococcal biofilms is adequate. More important barriers include the low metabolic activity and stress-adapted physiology of embedded bacteria, intracellular reservoirs, and poor vascular delivery across damaged or fibrotic tissue (Barman et al., 2024; Almatroudi, 2025). These mechanisms explain why antimicrobial therapy alone is often insufficient in chronic implant infection and why source control remains central.

The high rate of recurrence finds its explanation here. The model clarifies the propensity for relapse. Incomplete disruption of any node in the cycle can reignite the entire process (Kunnath et al., 2024). Residual biofilm fragments shelter within bone cell sanctuaries or microcalcifications. They can resuscitate once therapeutic pressure is lifted (Piuzzi et al., 2024). The persistently altered bone remodeling program and locally imprinted immunosuppressive microenvironment facilitate rapid re-establishment of the cycle. This occurs from a minimal residual bacterial burden. Clinical recurrence follows months or even years after the initial treatment (Kunnath et al., 2024).

Progressive osteolysis and aseptic loosening mimicry are directly addressed. The continuous operation of Phase 1 provides a mechanism for relentless periprosthetic osteolysis observed radiographically. This bone loss is an active, driven component of the infection’s life cycle. It is sustained by bacterial signaling and dysregulated host inflammation. It often occurs in the absence of systemic symptoms. It mimics aseptic loosening.

The BBI framework therefore complements, rather than replaces, established clinical models. Its value lies in organizing testable interactions that may identify adjunctive targets, such as excessive osteoclast signaling, matrix remodeling, local acidosis, or myeloid-cell dysfunction. Any cycle-directed therapy must be evaluated alongside surgical source control and guideline-based antimicrobial treatment, with claims calibrated to the level of evidence.

## 8 Translational perspectives: novel therapeutic strategies targeting the interface dialogue

### 8.1 Diagnostic frontiers: developing interface-specific biomarkers

PJI diagnosis currently relies on validated case definitions, including criteria developed by the Musculoskeletal Infection Society, the International Consensus Meeting, and the European Bone and Joint Infection Society (Parvizi et al., 2011; McNally et al., 2021). These frameworks integrate clinical findings, synovial-cell counts, microbiology, histology, and selected biomarkers. Diagnostic uncertainty remains in culture-negative infection, low-virulence organisms, early postoperative presentations, and differentiation from aseptic loosening or metallosis. Interface-specific biomarkers should therefore be investigated as adjuncts to established criteria, not as replacements for them.

Synovial fluid metabolomics reveals distinct metabolic reprogramming in PJI. Elevated lactate, acetate, and 3 hydroxybutyrate appear alongside decreased glucose and creatine. These changes reflect the hypoxic, glycolytic microenvironment. They also indicate overflow metabolism of pathogens such as *S. aureus*. Such metabolites offer a potential biomarker panel for early detection. Pathogen specific diagnosis becomes feasible (de Paula Mozella et al., 2024). Cytokine and chemokine arrays provide complementary information. Synovial fluid or periprosthetic tissue sampling captures dysregulated immune dialogue at the interface. Markers such as IL-6, IL-8, and procalcitonin already support the clinical distinction of PJI from aseptic failure, and more comprehensive profiling may extend this principle to reveal pathogen or biofilm specific immune fingerprints. Differential diagnosis would improve. Treatment response monitoring would become more precise.

Molecular imaging may add spatial information, but specificity remains limited because sterile inflammation can resemble infection (Al Mohajer and Darouiche, 2014; Johnson et al., 2025). Metabolomic, cytokine, microbial-DNA, and imaging signatures should be evaluated against established case definitions in clinically representative cohorts. Their most plausible role is to support difficult culture-negative or low-virulence cases rather than to redefine routine diagnosis prematurely.

### 8.2 Therapeutic avenues targeting the biofilm-niche interface

Emerging BBI-directed therapies should be framed as adjuncts to debridement, appropriate implant management, and antimicrobial therapy. Their proposed targets include the local microenvironment, bacterial metabolism, immune dysfunction, excessive bone resorption, and implant-surface behavior. Because evidence ranges from *in vitro* proof-of-concept to animal-model efficacy, translational readiness is graded explicitly in **Table 3**. These investigational strategies and their relationship to established standard care are summarized in **Figure 4**.

**Table 3:** Grading the translational readiness of therapeutic strategies targeting the BBI.

Strategy	Representative approach	Highest current evidence tier	Key translational gap	Reference
Microenvironment remodeling	Ultrasound-responsive hydrogel microspheres loaded with EGCG and MoS_2_ nanoparticles	Animal model efficacy (murine osteomyelitis)	No large animal or human data; manufacturing and ultrasound delivery standardization unresolved	(Steinmann et al., 2013; Murugan et al., 2024; Chen et al., 2025; Tian et al., 2026; Yin et al., 2026)
Metabolic intervention: quorum sensing inhibition	5-FU repurposing as an AI-2 quorum quencher	Animal model efficacy (murine MRSA biofilm infection)	Bone specific validation limited; safety window at infection sites undefined	(Fleitas Martínez et al., 2019; Sedlmayer et al., 2021)
Metabolic intervention: phytochemical combinations	Azithromycin plus kaempferol	Animal model efficacy (osteomyelitis models)	Pharmacokinetic interactions and optimal dosing in humans unknown	(Gao et al., 2021; Salehi Namini et al., 2025)
Immune modulation	MDSC-targeted therapies, phosphodiesterase-5 inhibitors, and checkpoint blockade	*In vitro* proof of concept and preclinical repurposing rationale; agents established for other indications	No controlled trials in infected orthopedic patients; risk of exacerbating inflammation or impairing healing	(Serafini et al., 2006; Chang et al., 2013; Ka et al., 2025)
Adjunctive bone protection	Short-term local bisphosphonate delivery via coated implants or carriers	Early clinical evaluation for implant fixation and osteoporosis; anti infective use investigational	Potential to promote bacterial adhesion and impair phagocytosis; infection specific trials lacking	(Li et al., 2010; Ganapathy et al., 2012; Kos et al., 2013; Jakobsen et al., 2016; Ren et al., 2023)
Smart targeted delivery	Bone-targeting conjugates (BV600022 and bisphosphonate-sitafloxacin)	Animal model efficacy (murine implant-associated osteomyelitis)	Human pharmacokinetics, linker stability, and long-term bone safety unproven	(Sedghizadeh et al., 2017; Ren et al., 2023)
Environment responsive coatings	pH-sensitive, enzyme-triggered, and ROS-responsive systems	*In vitro* proof of concept with limited *in vivo* data	Durability under physiological loading and long-term implant residence unvalidated	(Sedghizadeh et al., 2017; Yi et al., 2025; Liu et al., 2026; Zhang et al., 2026)

EGCG, epigallocatechin-3-gallate; MoS_2_, molybdenum disulfide; 5-FU, 5-fluorouracil; AI-2, autoinducer-2; MRSA, methicillin-resistant *Staphylococcus aureus*; MDSCs, myeloid-derived suppressor cells; ROS, reactive oxygen species.

**Figure 4:**
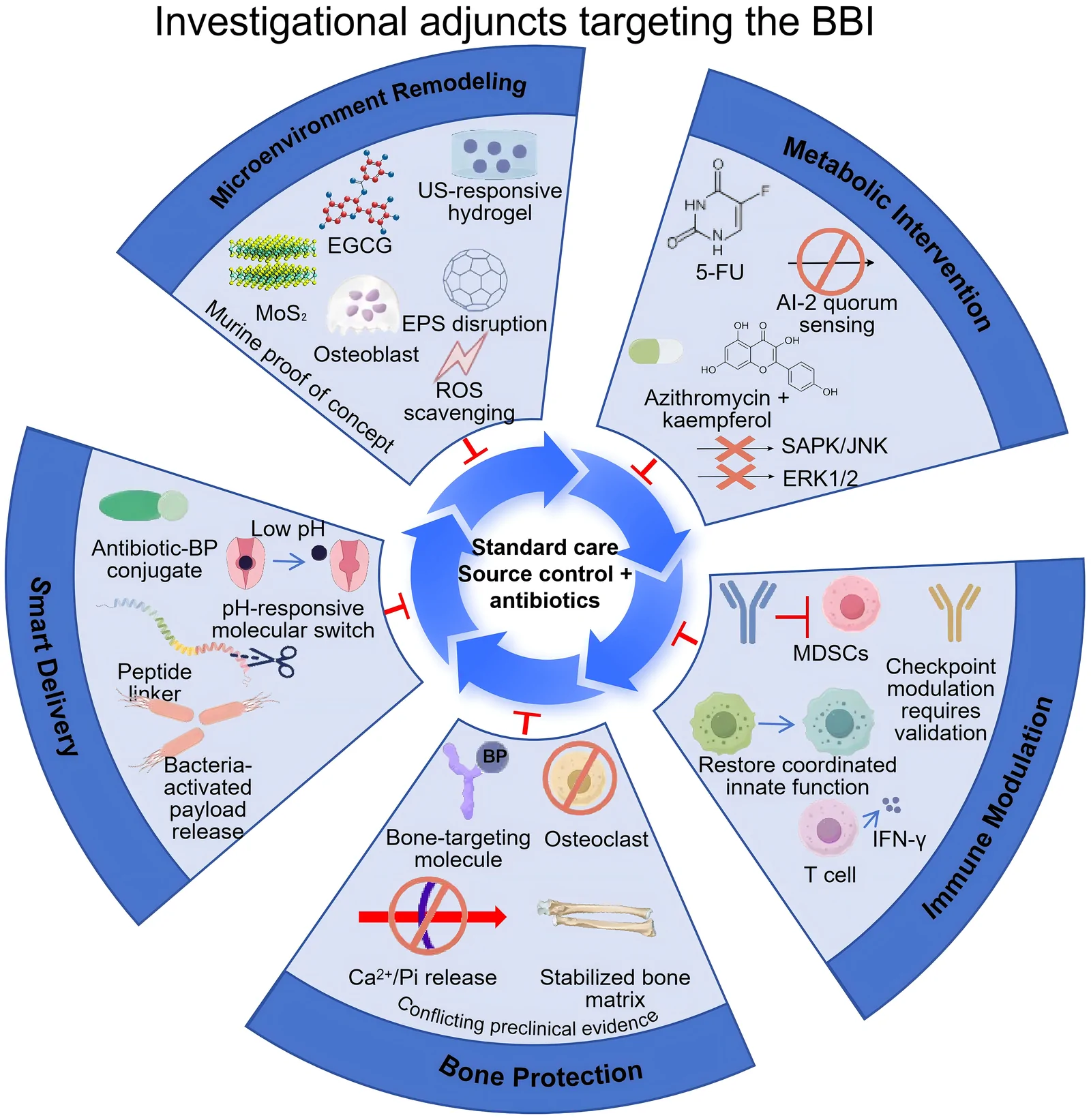
Investigational adjunctive strategies targeting the bone-biofilm interface. Surgical source control, appropriate implant management and targeted antimicrobial therapy remain the foundation of care. Investigational adjuncts include modulation of the hypoxic and acidic microenvironment, interference with biofilm metabolism and quorum sensing, restoration of coordinated innate immune function, limitation of excessive osteoclast activity, and stimulus-responsive local drug delivery. Representative preclinical approaches include ultrasound-responsive hydrogels containing epigallocatechin-3-gallate and MoS_2_, 5-fluorouracil-mediated interference with AI-2 signalling, azithromycin combined with kaempferol, targeting of myeloid-derived suppressor cells or checkpoint pathways, bone-targeted antibiotic conjugates, and pH- or bacteria-responsive delivery systems. These strategies are supported predominantly by *in vitro* or animal evidence and are not established alternatives to surgical source control or guideline-based antimicrobial therapy. Anti-resorptive approaches cannot restore the mechanical stability of a loosened infected implant. 5-FU, 5-fluorouracil; AI-2, autoinducer-2; BP, bisphosphonate; CTLA-4, cytotoxic T-lymphocyte-associated protein 4; EGCG, epigallocatechin-3-gallate; ERK1/2, extracellular signal-regulated kinase 1/2; MDSCs, myeloid-derived suppressor cells; PD-1, programmed cell death protein 1; ROS, reactive oxygen species; SAPK/JNK, stress-activated protein kinase/c-Jun N-terminal kinase.

#### 8.2.1 Remodeling the pathological niche: correcting hypoxia, acidosis, and ion imbalance

Acoustically responsive hydrogel microspheres illustrate a preclinical strategy for combined local delivery. In murine models, ultrasound-triggered release of epigallocatechin-3-gallate (EGCG) and MoS_2_ nanoparticles has been reported to disrupt biofilm, reduce oxidative stress, and support osteogenesis (Steinmann et al., 2013; Murugan et al., 2024; Chen et al., 2025; Tian et al., 2026; Yin et al., 2026). These findings establish proof of concept but do not yet demonstrate efficacy or safety in human implant infection.

#### 8.2.2 Metabolic intervention: precision targeting of biofilm nutritional dependencies and vulnerable pathways

Biofilms exhibit a distinct metabolic profile compared to their planktonic counterparts. QS coordinates behaviors and alters nutrient utilization pathways. These dependencies present attractive targets. Anti-virulence or biofilm sensitizing strategies aim to attenuate infection without applying strong bactericidal selective pressure (Fleitas Martínez et al., 2019). One promising avenue is QS inhibition. The interspecies signaling molecule autoinducer-2 (AI-2) serves as a key target. Li et al. (Sedlmayer et al., 2021) employed an innovative cell based screening platform in 2025. They repurposed the chemotherapeutic agent 5-fluorouracil (5-FU) as a potent quorum quencher. By inhibiting AI2 production in methicillin-resistant *Staphylococcus aureus* (MRSA), 5-FU disrupted biofilm formation and bacterial adherence. These effects occurred at concentrations significantly below its chemotherapeutic range. Crucially this quenching effect restored susceptibility of biofilm embedded MRSA to the antibiotic daptomycin *in vivo*. Metabolic interference can break biofilm mediated tolerance.

Synergistic combinations of antibiotics with phytochemicals target biofilm metabolism from another angle. Gao et al. (2021) showed in 2021 that combining azithromycin with the flavonoid kaempferol effectively attenuated azithromycin-resistant *Staphylococcus aureus* (ARSA) induced osteomyelitis. Beyond kaempferol’s direct anti-biofilm activity and antioxidant properties, the combination uniquely suppressed phosphorylation of key signaling proteins. Extracellular signal-regulated kinase 1/2 (ERK1/2) and SAPK/JNK were both affected (Salehi Namini et al., 2025). This suggests the therapy interfered with critical stress and survival pathways. Host cells activate these pathways. Infecting bacteria within the niche potentially exploit them. The metabolic adaptation crucial for persistent infection was compromised.

Together, these studies suggest that QS and metabolic adaptation are experimentally tractable targets. Their clinical value remains uncertain, and any future application would be adjunctive to source control and established antimicrobial therapy.

#### 8.2.3 Immune modulation: reversing immune cell dysfunction and lifting immunosuppression

Chronic orthopedic implant infection is sustained by a state of profound immune dysregulation, characterized by the suppression of effector functions and the dominance of immunosuppressive cells. MDSCs are central to this immunosuppressive network (Heim et al., 2014). In biofilm-associated infections, MDSCs are systemically elevated and locally recruited to the bone-implant interface, where they inhibit T cell function and pro-inflammatory responses through mechanisms such as arginine depletion and IL-10 secretion, effectively shielding the biofilm from immune clearance (Heim et al., 2014).

Strategies that inhibit MDSC recruitment or suppressive function have shown activity mainly in cancer models and remain speculative in orthopedic infection (Serafini et al., 2006; Ka et al., 2025). Similarly, interventions targeting T-cell exhaustion or antibody quality require infection-specific validation. Because neutrophils and macrophages execute direct bacterial killing, immune modulation should aim to restore coordinated innate function without intensifying tissue-damaging inflammation.

Immune modulation is therefore a preclinical research direction rather than a clinical alternative to surgery or antibiotics. Its potential lies in correcting local immune dysfunction after adequate source control, but safety, timing, and patient selection remain unresolved.

#### 8.2.4 Adjunctive bone protection: a strictly adjunctive role for short-term anti-resorptive agents

The inflammatory and infectious milieu of periprosthetic osteomyelitis is characterized not only by biofilm proliferation but also by a profound disruption of normal bone remodeling, often tilting the balance toward excessive osteoclastic resorption (Granata et al., 2022). This pathological bone loss not only compromises implant stability but also perpetuates the infection cycle by exposing new bone surfaces for bacterial colonization and releasing sequestered bacteria and nutrients that can fuel biofilm growth.

Short-term, localized anti-resorptive therapy has been proposed as an adjunct to limit excessive osteoclast activity after source control (Li et al., 2010; Ganapathy et al., 2012). The rationale is to reduce pathological bone loss and the accompanying release of matrix-derived signals. This concept remains hypothetical in infected implants and should not be interpreted as a means of reversing established implant loosening.

Evidence for bisphosphonates (BPs) in infection is conflicting and predominantly preclinical. Potential benefits in reducing osteolysis must be weighed against effects on immune function, bone turnover, bacterial adhesion, and biofilm persistence (Kos et al., 2013; Ren et al., 2023). If investigated, localized and time-limited delivery after debridement may be more defensible than prolonged systemic exposure (Jakobsen et al., 2016). No anti-resorptive agent can restore the mechanical stability of a loosened infected implant; surgical revision remains necessary when stability is lost.

#### 8.2.5 Advanced biomaterials: environment-responsive smart coatings and local targeted delivery systems

Antibiofilm surface engineering includes passive and active approaches. Passive nano- or microstructuring and antifouling chemistries aim to reduce the area or molecular interactions available for bacterial attachment, whereas active coatings release or present antibiotics, metal ions, antimicrobial peptides, nitric oxide, or matrix-disrupting agents. Environment-responsive systems add conditional release triggered by acidity, enzymes, reactive oxygen species, or ultrasound. Bone-targeting antibiotic conjugates and dual-function coatings can also combine local antibacterial activity with osteogenic support (Koo et al., 2017; Yang et al., 2022). However, topographies that favor osseointegration may also increase bacterial attachment area, and continuously eluting coatings may lose activity, select for resistance, or impair host-cell compatibility. Most nano- and microstructured surfaces and antimicrobial coatings therefore remain at the *in vitro* or small-animal stage, with unresolved questions about durability under loading, release kinetics, toxicity, manufacturing reproducibility, and regulatory evaluation.

**Table 4** summarizes candidate interventions directed at different components of the BBI. Their common aim is to complement bacterial killing by modifying local conditions that support persistence. At present, these approaches constitute a research agenda, and durable clinical benefit has not been established.

**Table 4:** Therapeutic strategies targeting the BBI.

Strategy	Representative approach	Mechanism of action	Key benefit	References
Microenvironment remodeling	Ultrasound-responsive hydrogel microspheres loaded with EGCG and MoS_2_ nanoparticles	Physical disruption of biofilm EPS barrier via acoustic cavitation; ROS scavenging; promotion of osteogenic differentiation; correction of hypoxia and acidosis	Concurrent antibiofilm and osteogenic effects reported in murine models; human benefit unestablished.	(Steinmann et al., 2013; Murugan et al., 2024; Chen et al., 2025; Tian et al., 2026; Yin et al., 2026)
Metabolic intervention	QS inhibition using repurposed 5-FU; combination of azithromycin with kaempferol	Blockade of AI-2 signaling; suppression of biofilm formation and bacterial adherence at sub-chemotherapeutic concentrations; inhibition of ERK1/2 and SAPK/JNK phosphorylation	Biofilm susceptibility and stress signaling altered in preclinical studies; clinical efficacy unestablished.	(Gao et al., 2021; Li et al., 2025; Salehi Namini et al., 2025)
Immune modulation	MDSC-targeted therapies; immune checkpoint inhibition; macrophage reprogramming	Reversal of local immunosuppression; promotion of MDSC differentiation into non-suppressive phenotypes; restoration of T cell effector functions; shift from M2 to M1 macrophage polarization	Potential restoration of coordinated immune function; infection-specific efficacy and safety unestablished.	(Serafini et al., 2006; Chang et al., 2013; Ka et al., 2025)
Bone protection	Localized short-term BP delivery via coated implants or biomaterial carriers	Suppression of pathological osteoclast activity via farnesyl pyrophosphate synthase inhibition; reduction of Ca^2+^/Pi release from bone matrix; transient “calming” of hyper-resorptive state	Potential reduction of pathological bone loss as an adjunct; cannot restore a loosened implant.	(Li et al., 2010; Ganapathy et al., 2012; Kos et al., 2013; Jakobsen et al., 2016; Ren et al., 2023)
Smart delivery	Bone-targeting antibiotic conjugates; pH-sensitive coatings; enzyme-responsive systems	pH-sensitive drug release at infection site; BP-mediated binding to hydroxyapatite at sites of high turnover; pathogen-specific triggered release	Higher local exposure in experimental systems; human pharmacokinetics, toxicity, and implant performance unproven.	(Koo et al., 2017; Sedghizadeh et al., 2017; Yang et al., 2022; Ren et al., 2023)

EGCG, epigallocatechin-3-gallate; MoS_2_, molybdenum disulfide; EPS, extracellular polymeric substances; ROS, reactive oxygen species; QS, quorum sensing; 5-FU, 5-fluorouracil; AI-2, autoinducer-2; ERK1/2, extracellular signal-regulated kinase 1/2; SAPK/JNK, stress-activated protein kinase/c-Jun N-terminal kinase; MDSC, myeloid-derived suppressor cell; BP, bisphosphonate; Ca^2+^, calcium ion; Pi, inorganic phosphate.

#### 8.2.6 Translational readiness of BBI targeted strategies

As summarized in **Table 3**, no BBI-directed cycle-breaking strategy is established for routine treatment of orthopedic implant infection. pH- or enzyme-responsive systems and several quorum-sensing approaches remain largely at the *in vitro* proof-of-concept stage (Sedghizadeh et al., 2017; Fleitas Martínez et al., 2019; Sedlmayer et al., 2021; Yi et al., 2025; Liu et al., 2026; Zhang et al., 2026). Ultrasound-responsive hydrogels, selected drug combinations, and bone-targeting antibiotic conjugates have shown efficacy in murine models (Steinmann et al., 2013; Sedghizadeh et al., 2017; Gao et al., 2021; Ren et al., 2023; Murugan et al., 2024; Chen et al., 2025; Salehi Namini et al., 2025; Tian et al., 2026; Yin et al., 2026). Repurposed agents such as BPs or phosphodiesterase-5 inhibitors are clinically available for other indications but have not been validated as adjuncts in infected orthopedic populations (Serafini et al., 2006; Ganapathy et al., 2012; Ka et al., 2025). Translation is limited by important differences between murine osteomyelitis models and human implant infection (Niska et al., 2012; Pribaz et al., 2012; Spellberg and Lipsky, 2012; Seok et al., 2013; Bronte et al., 2016; Carli et al., 2017; Billings and Anderson, 2022). Murine cortical structure, remodeling kinetics, immune physiology, implant geometry, mechanical loading, and antimicrobial pharmacokinetics differ substantially from those in patients. Efficacy in mice should therefore be interpreted as proof of mechanism, followed by validation in clinically relevant large-animal models and carefully designed human trials before routine use can be considered (Billings and Anderson, 2022; Peterlin et al., 2026).

#### 8.2.7 Limitations of the BBI model

While the BBI paradigm provides a unifying framework for understanding orthopedic implant infections, several important limitations must be acknowledged to ensure scientific rigor and guide future research directions.

Polymicrobial infection is a major limitation of the current BBI framework because most mechanistic evidence derives from monomicrobial *S. aureus* models. Mixed-species communities may alter metabolic exchange, immune stimulation, bone-cell responses, and treatment susceptibility in ways that cannot be inferred by adding single-species effects (Gabrilska and Rumbaugh, 2015; Dhamgaye et al., 2016; Abedon, 2019). Future studies should use clinically representative polymicrobial models and culture-independent profiling while distinguishing PJI, FRI, and other deep SSI populations.

Uncertain Long-Term Immune Memory Effects. The BBI framework emphasizes local immune dysregulation and myeloid cell dysfunction during active infection, yet the long-term immunological consequences of BBI establishment remain unclear. Whether the immunosuppressive microenvironment induces durable epigenetic modifications in tissue-resident immune cells, or whether “trained immunity” or “immune paralysis” persists after bacterial eradication, has not been systematically investigated (Schilcher and Horswill, 2020). Sepsis survivors exhibit prolonged myelopoiesis with expansion of MDSCs and subsequent long-term changes in myeloid functions months after the acute phase, suggesting that severe infections can induce lasting hematopoietic reprogramming (Schlüter et al., 2025). In the context of osteomyelitis, increased frequencies of regulatory T cells and B cells with elevated exhaustion markers have been observed, potentially facilitating pathogen establishment of chronicity (Sun and Barreiro, 2020). Such immunological memory could influence susceptibility to reinfection, wound healing capacity, and responses to subsequent surgical interventions. Additionally, the potential for biofilm-derived antigens to trigger autoimmune responses against bone matrix components warrants exploration. Longitudinal immunological studies in patients following successful treatment of implant-associated infections are needed to address these knowledge gaps.

Methodological Standardization Needs. Another limitation lies in the lack of standardized methodologies for BBI characterization. Variations in biofilm growth conditions, bone cell isolation protocols, and infection models across laboratories complicate cross-study comparisons and meta-analyses (De Zuani et al., 2026). The host cell to bacterial cell ratio, host EPS versus bacterial EPS volume, and number of bacterial cells encompassed within the biofilm are all important parameters that undoubtedly affect bacterial behavior, yet these are rarely standardized across studies (Surendar et al., 2024). Establishing consensus guidelines for BBI experimental design, biomarker assessment, and data reporting would enhance reproducibility and accelerate translational progress.

The BBI framework is therefore best regarded as a structured, testable synthesis with important evidentiary limits. Its refinement requires human translational studies, clinically representative polymicrobial models, standardized experimental methods, and longitudinal assessment of local immune and bone responses.

## 9 Conclusion

Orthopedic implant infection cannot be reduced to bacterial colonization of an inert surface. Established work has long recognized interactions among implants, biofilms, inflammation, and bone. The BBI framework does not claim originality for that general concept; it provides a structured way to organize these interactions, define evidence boundaries, and formulate specific testable mechanisms.

The principal contribution of the framework is threefold. It integrates dispersed observations within one spatial and functional interface, identifies candidate mechanisms such as ion-mediated matrix remodeling and osteoclast-associated bacterial persistence, and generates experimentally falsifiable questions. These propositions must be tested independently and should not be presented as established clinical drivers.

The model also emphasizes that persistence and recurrence arise from interacting processes, including bacterial physiological tolerance, intracellular reservoirs, inflammatory bone resorption, impaired innate clearance, and fibrotic or poorly vascularized tissue. This perspective explains why antibiotics alone may fail even when drug penetration into the biofilm is adequate.

Clinical management must continue to prioritize timely diagnosis, surgical source control, appropriate implant retention or exchange, and targeted antimicrobial therapy. Strategies directed at the microenvironment, metabolism, immunity, bone resorption, or biomaterials remain investigational adjuncts whose timing and safety require rigorous evaluation.

By separating established clinical practice from preclinical hypotheses, the BBI framework can guide research without overstating translational readiness. Its value will depend on whether future studies demonstrate that disrupting specific host-microbe interactions improves outcomes beyond current standard care.

## Glossary

5-FU: 5-fluorouracil
AI-2: autoinducer-2
ANR: anaerobic nitrate respiration regulator
ARSA: azithromycin-resistant *Staphylococcus aureus*
ATP: adenosine triphosphate
ArcD: arginine deiminase pathway D
BBI: bone-biofilm interface
BMP: bone morphogenetic protein
BMP2: bone morphogenetic protein 2
BP: bisphosphonate
COL1A1: collagen type I alpha 1 chain
CO₂: carbon dioxide
CTLA-4: cytotoxic T-lymphocyte-associated protein-4
Ca²⁺: calcium ion
DNR: dissimilative nitrate respiration regulator
EGCG: epigallocatechin-3-gallate
EPS: extracellular polymeric substance
ERK1/2: extracellular signal-regulated kinase 1/2
Eap: extracellular adherence protein
FcγR: Fcγ
FnBP: fibronectin-binding protein
HIF-1α: hypoxia-inducible factor-1α
ICL: isocitrate lyase
IFN-γ: interferon-γ
IL-10: interleukin-10
IL-12: interleukin-12
IL-1β: interleukin-1β
IL-6: interleukin-6
IcaB: intercellular adhesin B
MDSC: myeloid-derived suppressor cell
MMP: matrix metalloproteinase
MRSA: methicillin-resistant *Staphylococcus aureus*
Mg²⁺: magnesium ion
MoS₂: molybdenum disulfide
MyD88: myeloid differentiation primary response 88
NF-κB: nuclear factor-κB
NFATc1: nuclear factor of activated t cells cytoplasmic 1
NK: natural killer
OPG: osteoprotegerin
Opp: oligopeptide permease
PAMP: pathogen-associated molecular pattern
PD-1: programmed death-1
PIA: polysaccharide intercellular adhesin
PJI: periprosthetic joint infection
Pi: inorganic phosphate
QS: quorum sensing
RANKL: receptor activator of nuclear factor-κB ligand
ROS: reactive oxygen species
RUNX2: Runt-related transcription factor 2
SAPK: stress-activated protein kinase
JNK: c-Jun N-terminal kinase
SCV: small-colony variant
Sost: sclerostin
T3SS: type III secretion system
TGF-β: transforming growth factor-β
TLR2: Toll-like receptor 2
TLR4: Toll-like receptor 4
TNF: tumor necrosis factor
TNF-α: tumor necrosis factor-α
TNFR1: tumor necrosis factor receptor 1
TRAF6: tumor necrosis factor receptor-associated factor 6
TRAIL: tumor necrosis factor-related apoptosis-inducing ligand
VBNC: viable but non-culturable
Wnt: wingless-type
agr: accessory gene regulator
c-di-GMP: cyclic di-GMP
eDNA: extracellular DNA
*S. aureus*: *Staphylococcus aureus*
*P. aeruginosa*: *Pseudomonas aeruginosa*
*S. epidermidis*: *Staphylococcus epidermidis*
*E. coli*: *Escherichia coli*
*E. faecalis*: *Enterococcus faecalis*
CNS: Coagulase-negative staphylococci
FRI: fracture-related infection
SSI: surgical site infection
